# The effect of behavioural interventions targeting hand hygiene practices among nurses in high-income hospital settings: a systematic review

**DOI:** 10.1186/s40985-020-00141-6

**Published:** 2020-12-07

**Authors:** Madeline Sands, Alexander M. Aiken, Oliver Cumming, Robert Aunger

**Affiliations:** 1 Department of Infectious Disease, London School of Hygiene and Tropical Medicine, London, UK; 2 University of Arizona College of Medicine, Tucson, AZ USA; 3 Department of Clinical Research, London School of Hygiene and Tropical Medicine, London, UK

## Abstract

**Background:**

Hand hygiene is a critical behaviour for infection control but efforts to raise compliance among clinical professionals have been met with mixed success. The aim of this systematic review was to identify the effectiveness of the behaviour change techniques utilised in recent hand hygiene interventions that seek to improve hand hygiene compliance among nurses in hospitals in high-income countries. Nurses are at the frontline of healthcare delivery, and so improving their HH behaviour and thus increasing HHC rates will have a relatively large impact on reducing transmission and preventing healthcare acquired infections.

**Methods:**

High-quality studies among nurses in high-income countries were surveyed from the scientific literature, following PRISMA guidelines, to identify which kinds of behaviour change mechanisms have been used to effectively increase hand hygiene compliance. Only seven studies met all inclusion criteria. A formal meta-analysis was not conducted due to the heterogeneity of the included studies. Instead, the review analysed studies in line with the Intervention Component Analysis approach to identify which differences in intervention characteristics appear to be important. Analysis proceeded in two steps: first, the Effective Practice and Organization of Care Data Extraction Checklist was used to identify the study design and to describe the intervention, target population, setting, results, outcome measures, and analytic approach. The second step involved inferring the behavioural change techniques used in the complex study interventions. Following coding, logic models were then inferred for each study to identify the Theory of Change behind each intervention. These Theories of Change were then examined for suggestions as to which BCTs were likely to have been responsible for any effectiveness observed.

**Results:**

*Goals and planning* (to achieve specific ends), *comparison of behaviour* (to peers or some ideal) and *feedback and monitoring* (observing and providing feedback about behaviour or outcomes) were the most frequently used behaviour change technique groupings used across studies and within interventions.

**Conclusion:**

The complexity of the interventions used and lack of sufficient studies makes assignment of responsibility for behaviour change to specific behaviour change techniques difficult. Delivery channels and activities identified in the study Theories of Change were also highly individualized and so difficult to compare. However, we identified a temporal shift in types of techniques used in these recent studies on HH interventions, as compared with studies from prior to the review period. These newer interventions did not focus on providing access to alcohol-based hand rub or trying to solely encourage administrative support. Instead, they had nurses create goals and plan how to best facilitate HH, compared both individuals’ and the group’s behaviour to others, and focused on providing feedback.

**Supplementary Information:**

**Supplementary information** accompanies this paper at 10.1186/s40985-020-00141-6.

## Introduction

### Hand hygiene in the healthcare setting

Healthcare-associated infections (HAIs) burden patients, increase the length of hospital stays, raise the costs incurred by patients and healthcare facilities, affect treatment, and can lead to mortalit y[1–3]. Adequate hand hygiene (HH) among healthcare workers (HCWs) is considered to be the simplest and most effective measure for preventing HAIs [4]. However, observed practice of recommended HH behaviours among HCWs suggests that rates of compliance are typically below 50% [2, 5]. There have been various initiatives seeking to address these low rates of hand hygiene compliance (HHC) over the past several decades with mixed results.^1^ While many of these initiatives have been successful in producing short-term changes in compliance, the effects are typically small-to-moderate and sustained increased is low [1, 2].

Many HH interventions introduced in hospital-settings target multiple types of HCWs. However, rates of HHC have been shown to vary amongst the different healthcare professions; nurses have the highest compliance rates as compared to other HCWs [5, 6] Research has even shown that HCWs can respond differently to the same intervention [7, 8]. These results suggest that a “one-size-fits-all” strategy to hospital-wide education and quality improvement may not be the best strategy [7]. While interdisciplinary collaboration in hospital care is normative in current practice [9], it is nurses who have the most direct physical contact with patients within the healthcare delivery team [10]. As nurses are on the frontline of patient care, improving their HH behaviour and thus increasing HHC rates has a relatively large impact on reducing transmission and preventing HAIs. This review therefore concentrates on HH interventions designed specifically for nurses.

### Categorising and evaluating HH Interventions

HH is a complex behaviour influenced by varying combinations of individual, social, and environmental factors [11]. Multifaceted intervention strategies combining multiple components have been found to be more effective in addressing low compliance rates as compared to strategies focused on simple interventions [2, 12]. However, it can be difficult to assess which intervention components within multifaceted strategies contributed to changes in the observed behaviour and to what extent. Understanding how individual components have contributed to changes in HHC may support the development of more effective strategies.

In recent years, within the public health systematic review literature, there has been an increased focus on categorising and assessing interventions based on either the Theory of Change or behavioural frameworks used [13–17]. There are two systematic reviews—Huis et al. [2] and Srigley et al. [18]—that selected hospital-based HH interventions informed by behaviour change frameworks. Each review classified behaviour change interventions in different ways. Huis used Abraham and Michie’s (2008) [19] behaviour change technique (BCT) 1 taxonomy (which has since been updated) [20] while Srigley categorized interventions based on psychological theories of behaviour change. Both of these reviews identified successful strategies toward changing HH behaviour and, in doing so, have emphasized the importance of understanding how these strategies worked.

Objectively evaluating complex interventions is challenging [21], and various approaches such as Qualitative Comparative Analysis (QCA) and Intervention Component Analysis (ICA) have been recently employed in systematic reviews to understand the mechanisms through which different interventions attempt to change behaviour [17, 22, 23]. Here, we have adopted components of the ICA approach and created logic models to categorise and analyse interventions targeted at nurses.

## Methods

We report our methods in accordance with the Preferred Reporting Items for Systematic Reviews and Meta-Analyses (PRISMA) guidelines [24]. The protocol was pre-defined; the systematic review is not registered.

### Search strategy

Electronic searches were performed on three databases: MEDLINE, CINAHL, and EMBASE. The search strategy incorporated search terms related to the following:

1. Hand hygiene and hand washing,
2. Interventions, campaigns, and initiatives,
3. Compliance and adherence,
4. Hospital and healthcare setting,
5. Nurse and nursing

We also manually searched reference lists from five previous reviews for eligible studies: Gould et al. (2008) [25], Erasmus et al. (2010) [1], Huis et al. (2012) [2], Schweizer et al. (2013) [12], and Srigley et al. (2015) [18]. The search was first performed in August 2016 and then in October 2019. Search strings are included in Appendix 1-1.

### Eligibility and inclusion criteria

Only studies conducted in high-income countries (HICs)^2^ as per the World Bank’s 2016 definition and published in English were considered. Studies conducted between January 2002—when the CDC in the USA issued guidelines that defined ABHR as the standard of care for HH practices in healthcare settings [26]—and October 2019, were eligible for inclusion. In addition, only studies meeting all the following inclusion criteria were included in the review (Table 1):

1. The evaluated intervention targeted nurses and/or nursing students caring for patients in a hospital setting
2. The evaluated intervention focused on HH behaviours
3. The study clearly defined the intervention and had a control or comparison group; eligible study designs included cohort, case-control, controlled before-and-after, interrupted time series, cluster randomised trial, and randomised controlled trial
4. The study reported HHC rates as an outcome; rates could be measured by either direct observation or through indirect methods like calculating product usage or using an electronic monitoring system that counts sink or ABHR dispenser use
5. The study received a methodological quality score of three or greater

**Table 1:** Search criteria

	Inclusion	Exclusion
Date of publication	1 January 2002–22 October 2019	Before 1 January 2002 After October 2019
Location or context	Hospitals (e.g. ICU, medical wards, surgical units, inpatient units, entire facility) in high-income countries	All other settings; low-/middle-income countries
Intervention	Various forms of HH interventions	
Outcome	Measurements of observed improvement in HHC	Studies that do not measure improvement in HHC
Study design	Experimental: randomized-controlled trial (RCT) and non-RCT Experimental or quasi-experimental: pre-and-post intervention design with a control group; pre- and-post intervention design without a control group	Any other publications (e.g. outbreak reports, editorials)
Target population	Nursing staff; nursing student	Any other HCW

The studies were empirically rated on their level of quality using a rating system developed by Anderson and Sharpe (1991) [20] and adapted by Huis et al. (2012) [2] to evaluate the impact of interventions on either HCWs or patients (Table 2). Studies scoring less than three out of a possible seven points on the scale were considered of poor quality and excluded.

**Table 2:** Methodological quality rating

**Design of study**	Possible points
Experimental: randomized controlled trial (RCT), random allocation; case-controlled trial (CCT), quasi-random allocation; three data collection points before and after the intervention	1
Quasi-experimental: controlled before-and-after study; comparable control sites	1
Quasi-experimental: non-equivalent control sites	0
Single group before-and-after tests with baseline measurements	0
**Content**	
Intervention is clearly described	1
**Sample size**	
An *n* per group sufficient to detect a significant effect (*P* < 0.05) with a power of 0.80 or reported calculation of power	1
An *n* per group insufficient to detect a significant effect (*P* < 0.05) with a power of 0.80 or no reported calculation of power	0
**Validity and reliability of instruments**	
Unobtrusive observations, procedure described	2
Unobtrusive observations, procedure not described	1
Obtrusive observations, procedure described	1
Obtrusive observations, procedure not described	0
**Test statistics**	
Test statistics are described	1
**Significance**	
*P* value or confidence interval is given	1

### Article selection

Two reviewers independently screened the titles and abstracts of citations generated by the electronic and manual searches to assess their eligibility for consideration. AW and MHS reviewed the citations in the initial search in August 2016 and JS and MHS reviewed the citations in the updated search in October 2019. Any differences in selection were first resolved by consensus or, where this was not possible, by adjudication by a third reviewer (RA). Next, two reviewers (RA and MHS) independently reviewed the full-text articles to determine if the methodological quality criteria were met. The full text articles were then reviewed for inclusion by one reviewer (MHS).

#### Data extraction, synthesis, and analysis

A formal meta-analysis was not conducted due to the heterogeneity of the included studies across various parameters, including: content and delivery of the interventions, the moments during care for when HH performance was measured, and the methods for measuring the outcome variable and thus assessing compliance. Instead, the review combined qualitative and quantitative methods to analyse studies following the model of Intervention Component Analysis [23]. The ICA approach allows for the complete analysis of the individual components of each intervention without formal standard statistical technique to test the hypotheses. However, we only managed to implement certain aspects of the ICA approach. One departure from the standard procedure was the filtering of studies based on the quality of their research design, which is standard practice in quantitative systematic reviews. The ICA approach does not involve the evaluation of the methodological quality of studies. In addition, we created logic models for each intervention using BCTs to categorize and analyse the intervention components. The ICA approach sidesteps the creation of logic models. By combining logic models and components of ICA we adopted a comprehensive approach that facilitated the articulation of the theoretical basis of the interventions and identification of BCTs. In this way, the present review combined quantitative and qualitative methods of analysis.

We examined how the interventions differed from one another using a two-step approach. The first step used the Effective Practice and Organization of Care (EPOC) Data Extraction Checklist to identify the following characteristics in each study: (a) study design, (b) description of intervention, (c) target population, (d) setting, (e) results, (f) outcome measures, and (g) analysis. The second step involved inferring the BCTs, which informed the various activities in these complex interventions. We used the taxonomy of BCTs developed by Michie et al. (2013) [20] due to its standardised labels, clear definitions, and examples; also, this taxonomy is widely used among researchers, practitioners, and policy-makers. The taxonomy includes 93 BCTs clustered into the following 16 groups:

1. Goals and planning
2. Feedback and monitoring
3. Social support
4. Shaping knowledge
5. Natural consequences
6. Comparison of behavior
7. Associations
8. Repetition and substitution
9. Comparison of outcomes
10. Reward and threat
11. Regulation
12. Antecedents
13. Identity
14. Scheduled consequences
15. Self-belief
16. Covert learning

Two reviewers (MHS and RA) used the taxonomy to independently code the various intervention components in each study. Differences in coding were resolved by consensus. Following the coding, logic models were then inferred for each study by incorporating the nominated BCTs, activities and modes of delivery; this guided the development of the Theory of Change behind each intervention, which is based on the approaches used by Govender et al. (2015) [13] and Kahwati et al. (2016) [17]. To ensure that the models accurately reflected the Theory of Change hypothesised by the studies, the authors of each study were contacted and asked to review the logic model. Only Stock verified the Theory of Change; the other authors did not respond. Frequencies with which the BCT categories were implicated in the studies were then calculated and compared.

## Results

**Fig. 1:**
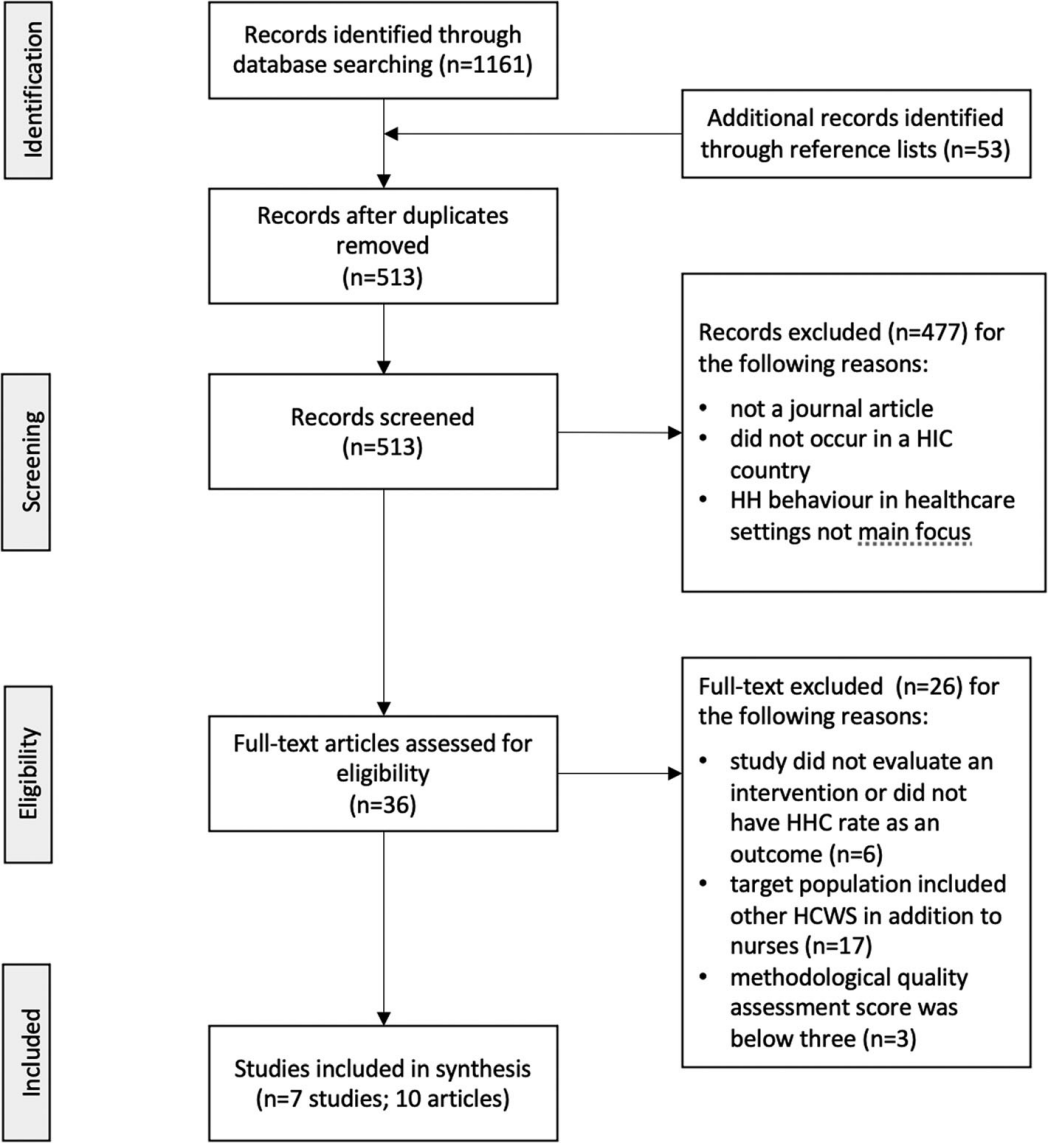
Flow diagram for study selection. The flow of information through the different phases of the systematic review is depicted including the number of records identified and then number included and excluded

A total of 1214 articles were identified across three databases and from reference lists of previous reviews (Fig. 1). After duplicates were removed, 513 records were screened of which 477 were excluded due to not being a journal article, not being conducted in a HIC, or not evaluating HHC rates as the main outcome. The full text of the remaining 36 articles were assessed for eligibility resulting in a total of 7 studies (10 articles)^3^ that met the inclusion criteria [27–34]. The three main reasons for exclusion of the other 26 articles were: (1) that the study did not evaluate an intervention (*n* = 6), (2) the target population of the intervention included other HCWs in addition to nurses and did not allow for separate analysis (*n* = 17), or (3) the methodological quality assessment score was below three (*n* = 3).

### Study characteristics

The seven studies included in this review are as follows:

- Fox et al. 2015 [30]
- Erasmus et al. 2010 [27]
- Stock et al. 2015 [28]
- Harne-Britner et al. 2011 [29]
- Huis et al. 2012 [31, 32, 35]
- Boyce et al. 2019 [33]
- Stella et al. 2019 [34]

Characteristics of the included studies are presented in Table 3.

**Table 3:** Study characteristics

**Characteristic**	**References**
**Setting**	
Intensive care unit (ICU)	Fox; Erasmus; Huis; Boyce
Medical unit	Stock; Huis
Surgical unit	Erasmus; Huis
Mixed medical-surgical unit	Stock; Harne-Britner; Boyce; Stella
Paediatric unit	Huis
Progressive care/step-down unit	Boyce; Stella
**Sample type (HCWs)**	
Nurses only	Erasmus; Fox; Stock; Huis; Boyce
Nurses and nursing assistants	Harne-Britner; Stella
**Sample size (HCWs)**	
< 20	Erasmus
20–40	---
41–60	Stock
> 60	Harne-Britner; Huis; Stella
Unknown	Fox; Boyce
**Sample size (observations)**	
< 100	
100–500	Erasmus; Harne-Britner
501–1500	Stock
1501–2500	---
2501–5000	---
> 5000	Huis; Boyce; Stella
Unknown	Fox
**Behavioural frameworks, theories, and approaches**	
Behaviourist Theory	Harne-Britner
Change Theory	Harne-Britner
Field Theory	Harne-Britner
Health Action Process Approach	Erasmus
Social Cognitive Theory	Harne-Britner; Huis
Social Norms (Behavioural Economics)	Stella
Toyota Kata	Boyce
Not listed	Fox, Stock
**Study design**	
Before-after	Stock; Fox; Erasmus
Case-control	Harne-Britner
Cluster randomized control	Huis
Quasi-experimental w/ interrupted time series	Boyce; Stella
**Assessment of compliance**	
Direct observation	Stock; Fox; Erasmus; Harne-Britner; Huis
Electronic monitoring system	Boyce; Stella
**Length of Study**	
> 6 months	---
6 months	Erasmus; Stella
12 months	---
14 months	Huis
15 months	Fox
16 months	Stock
> 2 years	Boyce
**Country**	
Europe	Stock; Erasmus; Huis
North America	Fox; Harne-Britner; Boyce; Stella
**Assigned methodological quality score**	
6	Erasmus; Harne-Britner; Boyce; Stella
7	Fox; Huis; Stock

### Hand hygiene intervention studies

**Fig. 2:**
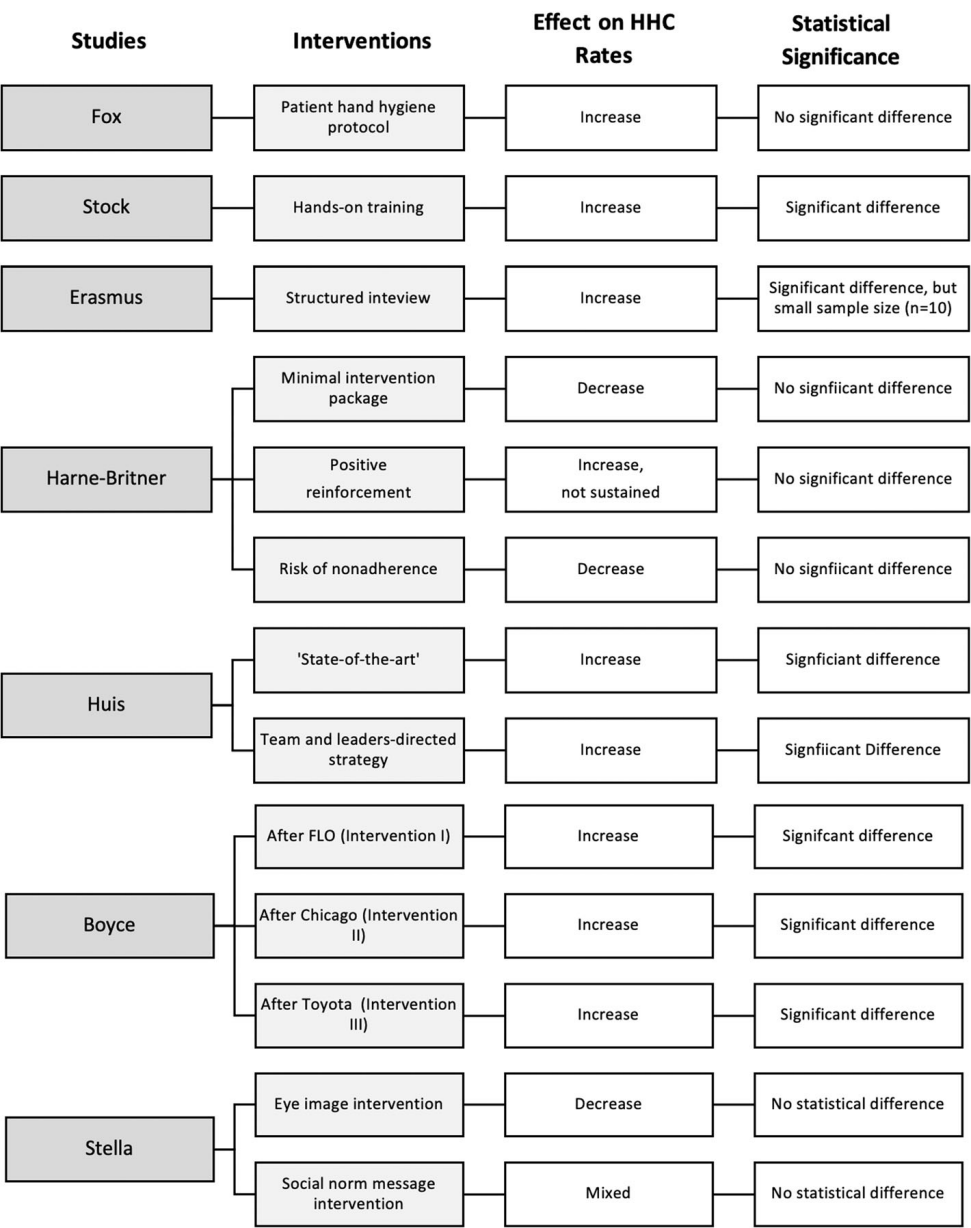
Summary of the studies included in the review

Three of the studies only evaluated one intervention while the other four studies reported on two or more interventions (Fig. 2). The studies and their intervention(s) are described below, based on the authors’ own descriptions. The Theories of Change (see Appendix 1-4) reflect the descriptions provided here.

Fox et al. (2015) [30] performed a pre-experimental (post-test only with a comparison group) study design comparing nurses’ HHC rates and the rates of two common HAIs—central line-associated bloodstream infections (CLABSI) and catheter-associated urinary tract infection (CAUTI)—before and during the intervention. The study was conducted in a cardiovascular medical ICU in a 498-bed community hospital in the USA from December 2009 to February 2012. The study involved three phases: (1) a comparison 12-month period before protocol implementation, (2) a 10-week protocol-training period, and (3) a 12-month period during the protocol implementation. The innovative characteristic of this intervention was focusing attention on the patient’s HH rather than on the HCW’s HH practices. Nurses were required to wash the patient’s hands three times a day: at 8am, 2pm, and 8pm. There was a 10-week protocol phase-in period in which training of the ICU staff was led by the study team. Nursing staff received verbal instructions and were monitored for proper return demonstration of the protocol in efforts to improve consistency of HH technique. In addition, the electronic medical record (EMR) triggered timely reminders to perform the patient hand hygiene protocol (PHHP). Nurses documented their own PHHP adherence on the EMR. During the execution phase, the primary ICU nurse introduced the PHHP to each patient and/or patient’s family; a document explaining the protocol was added to each ICU patient’s admission packet.

Nurses’ HHC when entering patients’ rooms increased from 35 to 66% during the study. Although there was an improvement, the difference was deemed not statistically significant. Nurses’ HHC when exiting the patient’s room also improved with an increase from 66 to 79%, but the results were not as remarkable.

Harne-Britner et al. (2011) [29] conducted a quasi-experimental (controlled before-after) study conducted among registered nurses and patient care assistants from three medical-surgical units at an urban hospital in the USA. It was conducted from April to October 2005. Both HHC and unit HAI rates were measured, with HH observations taken each month for 6 months (May to October 2005). The study was participants in the control group received HH education by completing a self-study module on handwashing. The intervention groups completed the same module but also received positive reinforcement (a sticker-reward system that included individual and unit rewards) or additional information on the risks on HH non-compliance. These were grounded in Control Theory, Social Cognitive Theory, Behaviourist Theory, and Field Theory. These two interventions were evaluated against the standard minimal intervention comparator group, which received basic HH education via a self-study module. This study was therefore considered to be assessing three different interventions.

After 1 month of the intervention, the HHC among the positive reinforcement group increased by 15.5% (*χ*^2^ = 4.27, *P* = 0.039), but decreased in the risk of non-adherence group (6.4% decline) and the control group (3.2% decline). While the positive reinforcement intervention initially improved HHC, this effect was not sustained throughout the study. By the sixth month, there were no significant differences in HHC or HAI rates between the three groups. *Harne-Britner* concluded that both the education-alone and the education-paired-with-negative-behaviour interventions did not result in sustained improvement of HHC. However, the peer-recognition and unit-reward programs paired with education were effective in producing an immediate increase in HHC rates; *Harne-Britner* argued that these approached could be effective in promoting long-term HHC.

Stock et al. (2016) [28] assessed the feasibility of an innovative hands-on training session aimed at improving HHC through a before-after controlled cohort trial. The study was conducted from October 2012 to March 2014 in a large university hospital in Germany with 50 trained nurses from three medical and medical-surgical units (gynaecology, neurology, and nephrology). HHC rates were measured, with a baseline covering a 12-week span pre-intervention and follow-up covering a 12-week span post-intervention. Content and form of the educational intervention were developed based on the German Institute for Hygiene and Infection Control’s current guidelines and the objective structured clinical examination (OSCE).^4^ The hands-on training was organized into four separate parts, which were delivered over one and half days of consecutive training. The first part focused on providing the research team with a baseline assessment of the participants’ hygiene skills while also giving participants the chance to reflect on their own hygiene and communication skills. Part two involved a learning session on communication skills related to promoting hygiene at the workplace. The session featured lectures, role-play, reflection, evaluation, quality management in hospital hygiene, and various methods to address barriers to hygiene when communication with peers and superiors. The third part centred on combining the theoretical with the practical in the form of simulation training. Participants practiced hygiene skills in different situations under the supervision of the infection control nurse. In the fourth and final part, the initial assessment was repeated to evaluate improvements in hygiene skills.

Overall HHC rates increased from 64.3% before the training to 79.2% after the training (*P* ≤ 0.0001). Stock identified two biases that could have attributed to the high compliance rates: (1) the Hawthorne effect (participants increased HHC because they were aware that they were being monitored) and (2) self-selection bias introduced by the “opt-in” design of the study. Despite the acknowledged possible biases, Stock concluded that monitoring, feedback, and implementation of teaching ‘on the job’ are effective tools in increasing HHC.

Erasmus et al. (2010) [27] explored the practicality and effects of action planning on HH behaviour of nurses in an ICU and surgical unit of a university teaching hospital in the Netherlands. This work was intended as a pilot study. A pre-post-test design, using the Health Action Process Approach [36], was conducted from March to August 2008. HHC rates were measured at baseline and then at 3 weeks post-intervention. The intervention consisted of a structured interview of around 30 min that covered the importance of HH, rated self-compliance, preferred methods of HH, and the possible barriers encountered in daily practice. Individualised action plans for performing HH were then made. In addition to action planning, participants had to anticipate and plan alternatives for moments when the situation did not lend itself to the facilitation of HH. No feedback was given regarding the correctness or quality of the participants’ action plans.

HHC rates increased from 9.3% at baseline to 25.4% post intervention (*P* < 0.001). Nurses were 3.3 times more likely to perform HH (odds ratio [OR]: 3,3; confidence interval [CI] 1.7–6.5]) after the intervention. Erasmus acknowledged numerous limitations of the study such as the small number of participants and the short time span between intervention and follow-up. Although considered a pilot study, Erasmus argues that action planning could feasibly be used as a change strategy through bridging the intention-behaviour gap and thus leading to improved HHC in practice. Yet, Erasmus recognizes that action planning is unlikely to have sufficient effects as a single intervention (the overall shift in compliance from 10% to 25% was far too low), and as such should be part of a multiple component intervention that addresses individual, social, environmental, and planning variables.

Huis et al. (2013) [31, 32, 35] tested whether a social cognitive theory-based team and leaders-directed strategy would be more effective in increasing HHC rates in nurses than a literature-based state-of-the-art strategy. A cluster randomised controlled trial was conducted between September 2008 and November 2009 in 67 nursing units of three hospitals in the Netherlands. Baseline data were collected right before intervention implementation. Interventions were delivered over a period of six months. Follow-up measurements were recorded directly after the strategy delivery and then at 6 months. The control arm received the ‘state-of-the-art’ strategy, which included (a) education for improving relevant knowledge and skills, (b) reminders for supporting the actual performance of HH, (c) feedback to provide insight into current behaviour and to reinforce improved behaviour, and (d) providing for adequate products and facilities. The team and leaders-directed strategy included all elements of the ‘state-of-the-art’ strategy (a–d) in addition to (e) gaining active commitment and initiative of unit management, (f) modelling by informal leaders at the unit, and (g) setting norms and targets within the team. This was therefore considered as two separate interventions.

The HHC rates of the state-of-the-art group increased from 23 to 42% in the short term and then to 46% in the long run. The HHC in the team and leaders-directed group improved from 20 to 53% in the short term and remained at 53% in the long term. The difference between both strategies showed an odds ratio of 1.64 (95% CI 1.33–2.02; *P* < 0.001) in favour of the team and leaders-directed strategy. *Huis* emphasize that their results support various behavioural science theories, which hold that social influence, team effectiveness, role modelling, and leadership are necessary to successfully change behaviour.

Boyce et al. (2019) [33] performed a retrospective, non-randomised, observational, quasi-experimental study in a single 93-bed non-profit hospital in the USA from August 2015 through January 2018. The study evaluated the installation of an automated HH monitoring system (AHMS) and three defined interventions: (1) a frontline ownership (FLO) initiative, (2) support by hospital leadership, and (3) implementation of a Toyota Kata methodology. The ‘FLO initiative’ involved an expert visiting the hospital on three separate occasions to assist in implementing FLO. The ‘support by hospital leadership’ intervention consisted of the hospital leadership sending a delegate to another hospital to learn about their successful multimodal HH campaign and to discuss methods for analysing AHHMS data and incorporating additional promotional activities. The third intervention, which adopted aspects of the Toyota Kata performance improvement methodology, encompassed mandatory trainings, staff members wearing a “sheriff” badge and reminding personnel to perform HH, daily reportings of HH rates during shift huddles, and coaching of HCWs when compliance rates decreased. The interventions were staggered across various hospital units.

Boyce found that installation of the AHHMS without supplementary activities did not yield sustained improvement in HHC rates. However, implementation of the three interventions resulted in a statistically significant 85% increase in HH performance rates (*P* < .0001). Boyce also looked at HAI rates and observed that the incidence density of non–C. difficile HAIs decreased by 56% (*P* = .0841), while C. difficile infections increased by 60% (*P* = .0533) driven by 2 of the 4 study units.

Stella et al. (2019) [34] studied the effect of two visual cues on HHC in a prospective, quasi-experimental study that utilised an interrupted time-series design. Intervention placards that depicted an image of eyes, a social norms message, or a control placard (image of mountains) were placed near soap and ABHR dispensers and alternated every 10 days. HH opportunities and compliance rates were assessed electronically over a 4-month study period. The pre-intervention baseline HHC rate was 70%. No statistically significant increase in HHC was observed as a result of either intervention.

### BCTs addressed

The HH intervention(s) from each study were broken down into their individual components and the BCTs utilised were coded accordingly (Table 4). Explanations for the coding of each study are given in 1-2 and the resulting Theories of Change are included in Appendix 1-3.

**Table 4:**
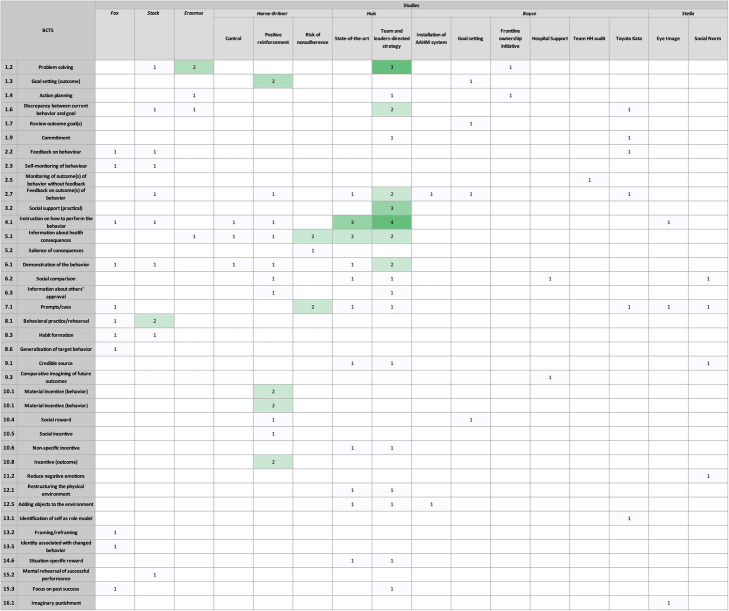
Distribution of BCT codes by study and intervention

**Fig. 3:**
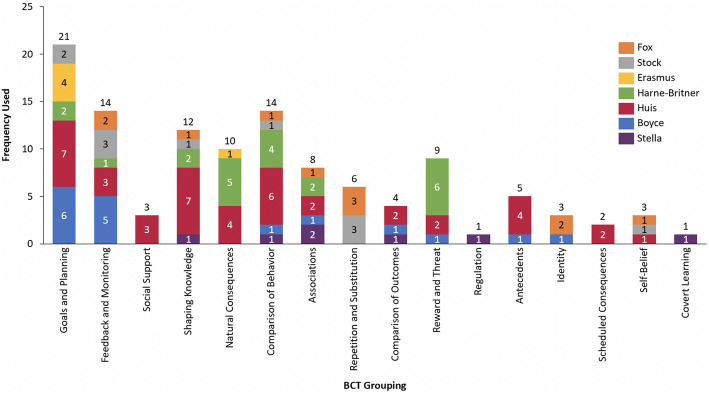
BCT groupings across studies

**Fig. 4:**
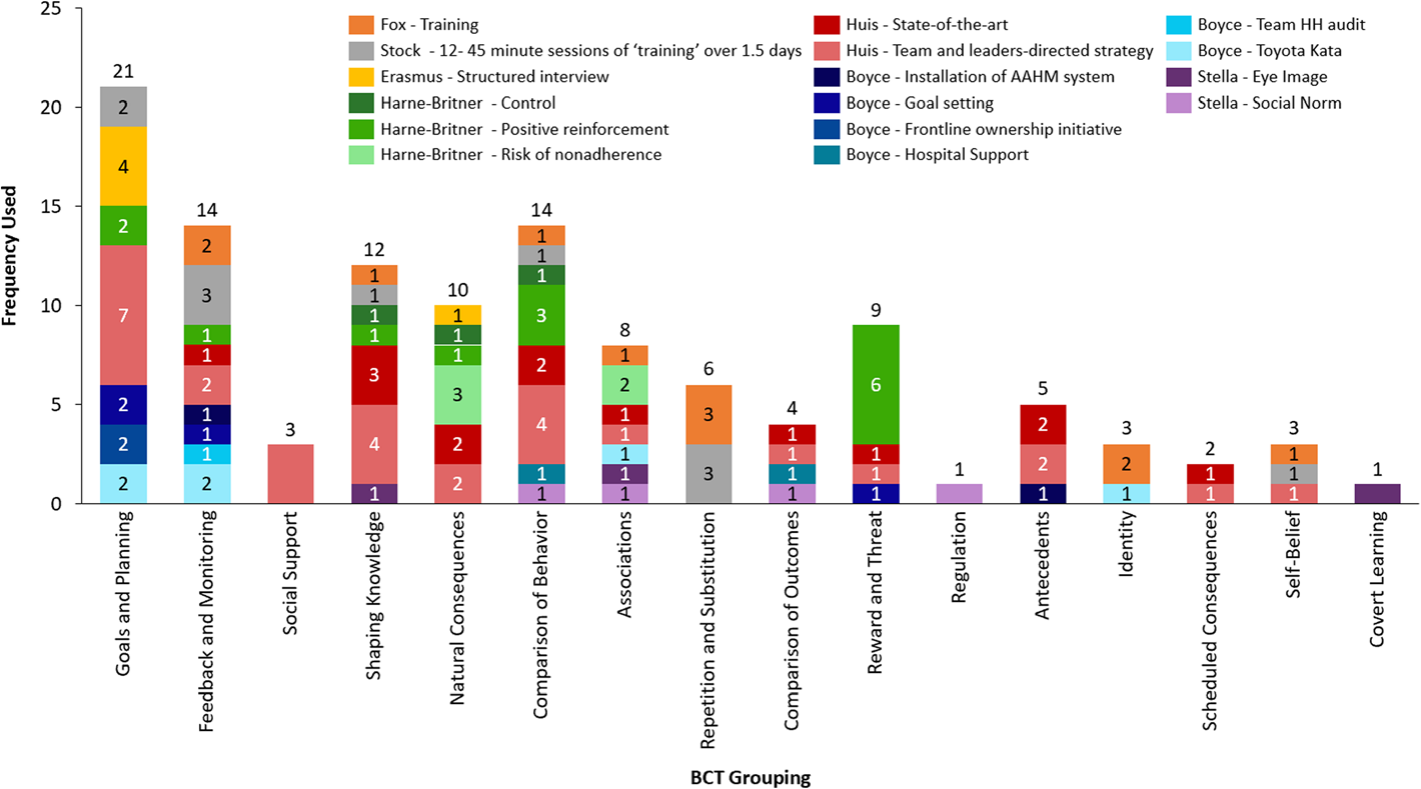
BCT groupings across interventions

Every BCT grouping was used across all studies. However, the BCT groupings *goals and planning*, *feedback and monitoring*, *comparison of behaviour*, and *shaping knowledge* were the most commonly used among the majority of studies and were most frequently used within interventions (Tables 2, 3, 4, and 5). As depicted in Fig. 3, BCTs from all 16 groups were used by at least one study in our sample. The most widely used groupings across studies were *comparison of behaviour* (*n* = 6 studies), *goals and planning* (*n* = 5 studies), *feedback and monitoring* (*n* = 5 studies)*,* and *associations* (*n* = 4 studies). When looking at BCTs across interventions, the BCT grouping that was most frequently used was *goals and planning,* which was coded 21 times across 6 studies, as seen in Fig. 4. However, of the 21 coded components, 7 of those belonged to *Huis’* team and leaders-directed strategy. The BCTs from other groupings that were more commonly used include: *feedback and monitoring* coded a total of 14 times across 9 interventions, *comparison of behaviour* coded 14 times across 8 interventions, and *shaping of knowledge* coded 12 times across 7 interventions.

**Table 5:**
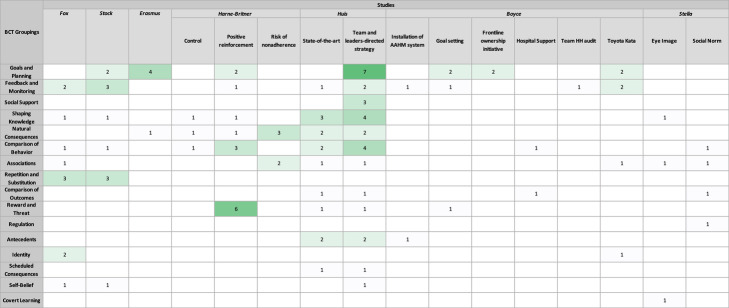
Distribution of BCT groupings by study and interventions

These groupings were used in different ways. In regard to *comparison of behaviour*, Stock used these BCTs in the form of having nurses compare their own HH practices to the simulation training demonstrations, Harne-Britner’s positive reinforcement sticker system served as another way to compare behaviour, and Stella’s social norm message placards prompted nurses to compare their behaviour to the HCWs on the placards. The g*oals and planning* grouping was used by Erasmus’ in their action and coping planning activities, in Boyce’s frontline ownership initiative where the hospital actively sought to create a solution for low HH rates, and in Huis’ team and leaders-directed strategy which used analysis of the barriers and facilitators to HH in order to help nurses’ with their own compliance. While *feedback and monitoring* was implemented in different ways, a common approach seen across interventions was reviewing HH rates with nurses during regularly scheduled meetings (as seen in Harne-Britner’s positive reinforcement intervention, in both of *Huis’* interventions, and throughout Boyce’s various strategies). The one grouping that consisted of the same BCT utilised across all studies and within interventions was *knowledge shaping*, in which instruction on how to perform HH was provided.

## Discussion

This review found that the BCT groupings *goals and planning*, *feedback and monitoring*, *comparison of behaviour*, and *shaping knowledge* were commonly utilised across a majority of studies. Moreover, BCTs from these groupings were also the most frequently used within the interventions. It should be noted that even though each BCT groupings was utilised across all studies, and while some groupings were significantly used, the actual techniques employed were limited. There were many techniques within each grouping that had not been addressed. For example, the *knowledge shaping* grouping is comprised of four techniques, yet all studies only incorporated the technique on instruction of behaviour (BCT 4.1: instruction on how to perform a behaviour). Thus, the relatively narrow range of actual techniques used within each grouping suggest that new campaigns could look to other, unused forms of promotion to achieve sustained improvements in HHC.

The three studies that produced statistically significant increases in HHC rates were Stock, Huis, and each of Boyce’s strategies sans the initial AHHMS approach. The four BCT categories common amongst these three studies included *comparison of behaviour*, *shaping of knowledge*, *feedback and monitoring*, and *goals and planning* (although this last BCT grouping was only present in Huis’ team and leaders-directed intervention).

These three studies were also among those that incorporated the most BCTs in each of their interventions. There has been discussion in the literature about the association between number of BCTs included and the effect on HHC rates. One review observed that the effect size of HH improvement increased when more BCTs were addressed [2]; another review did not see such a relationship between increase in effect size and number of BCTs included [12]. In this review, the three studies found to be associated with increased HHC each included more than five BCTs.

Unfortunately, due to the small number of studies which matched our inclusion criteria, the overlap between BCTs used in both effective and non-effective interventions, the small number of studies demonstrating a significant outcome, and the diversity of conditions of delivery and measurement, it simply is not possible to identify which BCTs are associated with a higher probability of improving nurse HHC.

The present analysis, however, does expand upon what previous reviews, conducted between 2002 and 2012, found. Those studies identified successful HH interventions as multifaceted approaches that bundle education, reminders, feedback, and in some cases access to ABHR and the inclusion of administrative support [2, 12]. This review, looking at publications between 2002 and 2019, identified a shift in the components incorporated in recent HH interventions. While most of the reviewed interventions included the conventional components of education, reminders, and feedback, many of these interventions included two additional components that had previously been underutilized: in particular, *comparison of behaviour* both at the individual and hospital unit level and *goal setting* for setting goals to reach certain HHC rates and creating plans to reach such goals. The comparison of behaviour activities, which are now being included in these interventions, draws attention to others’ performance, prompt nurses to imitate a certain behaviour, and highlight the social acceptance of HH. By having nurses devise and work towards a HH goal, the nurses become involved in a greater initiative—that they have decided upon—that establishes an expectation of the post-intervention outcome. Affiliation and self-empowerment serve as motivators for increasing HH practice. This shift in intervention components could be attributed to the date of publication of the considered research papers. Our inclusion criteria during the study selection process resulted in a pre-dominance of studies published within the past 10 years. In the present day, almost all hospitals in the USA and Europe provide ABHR at the point of patient care [5, 37, 38]. Ensuring that ABHR is readily accessible is no longer a main focus of current HH interventions in HIC hospital settings.

### Limitations

Several limitations must be acknowledged regarding our analytic approach, search criteria, sample size, determining of effectiveness, and the inherit bias and difficulties that arise in coding.

#### Eligibility and inclusion criteria

In searching for articles, we were limited by the language and location of studies. Only papers in English were included due to the authors’ own linguistic capacities. Thus, potential articles written in other languages were overlooked. Also, by only considering studies conducted in HIC, we excluded potential studies from low- to middle-income countries in highly resourced hospitals with infrastructure comparable to that in HIC.

#### Small number of papers

A rating system was used to evaluate relative methodological quality. Due to the lack of moderate to high-quality HH improvement studies, the review only considered seven studies. This review provides insight even if it reflects only a small number of papers because conclusions drawn from analysis of these papers are well founded as compared to papers of lower methodological quality.

#### Determining effectiveness

We were unable to calculate effectiveness for most of the studies due to three main reasons: (1) not every study had a control group, (2) the studies defined HH opportunities in different ways, and (3) measurements of HHC pre- and post-intervention were taken at different times for each study. By comparing the effectiveness or relative differences for each intervention, we would have been able to determine if a relationship existed between effectiveness and number of BCTs used. The limitations mentioned above are a few examples of methodological weaknesses. In fact, multiple systematic reviews have recognized that there are severe design limitations in various HH studies [2, 12, 25, 39].

## Conclusion and future directions

The purpose of this review was to identify the mechanisms, and the corresponding BCTs used, by which recent HH interventions sought to improve HH behaviour amongst nursing personnel. We used Intervention Component Analysis to guide our processes and analytic strategy. The specific improvement activities for each intervention were identified and classified using Michie’s BCTs taxonomy. This review underscores the importance of truly understanding how and why a change in behaviour—such as an increase in HH practice—is expected to happen in the particular context. Many studies cite behavioural frameworks yet fail to explain how the frameworks were operationalized and which BCTs were utilized. It can be argued that the real pitfall in these sorts of studies comes from the misunderstanding and mischaracterisation of HH behaviour. HH is a repetitive, automatic behaviour that is habit-forming [18, 40] However, many studies create interventions that treat HH as if it were a deliberative action rather than a spontaneous behaviour involving non-thoughtful responses that are shaped by the behaviour setting. It is simply not enough for interventions to be grounded in behaviour change theory; interventions must employ behaviour change theories and utilise BCTs that are appropriate for the type of behaviour at hand [18].

Previous reviews have indicated that successful HH interventions are multifaceted approaches that bundle education, reminders, feedback, and in some cases access to ABHR and the inclusion of administrative support. We identified a shift in types of techniques used in these more recent studies on HH interventions, as compared with studies from prior to the review period. These newer interventions did not focus on providing access to ABHR or trying to solely encourage administrative support. Instead, they had nurses create goals and plan how to best facilitate HH, compared both individuals’ and the group’s behaviour to others, and focused on providing feedback.

It has been difficult to draw inferences from complex interventions as to which aspects of the intervention were effective in creating the observed behaviour change, due to a number of limitations in the current literature. However, analysing interventions based on the BCTs employed offers insight into how the proposed mechanisms may have succeeded or failed in changing behaviour. We recommend that additional reviews be conducted in this manner once additional studies have been published.

## Supplementary Information

**Additional file 1.** Search strings

**Additional file 2.** Explanation of BCTS identified in studies

**Additional file 3.** Logic models developed for studies

## Data Availability

All data generated or analysed during this study are included in this published article and its supplementary information files.

## Abbreviations

ABHR: Alcohol-based hand rub
BCTs: Behaviour change techniques
CAUTI: Catheter-associated urinary tract infection
CDC: Centres for Disease Control
CLABSI: Central line-associated bloodstream infections
EMR: Electronic-medical record
EPOC: Effective Practice and Organization of Care
HAIs: Healthcare associated infections
HCWs: Healthcare workers
HH: hand hygiene
HIC: High-income country
ICA: Intervention Component Analysis
ICU: Intensive care unit
OSCE: Objective structure clinical examination
PHHP: Patient hand hygiene protocol
PRISMA: Preferred Reporting Items for Systematic Reviews and Meta-Analyses
QCA: Qualitative Comparative Analysis

## Footnotes

1 Behaviour change techniques (BCTs) are intervention mechanisms that target a specific determinant of a behaviour in order to trigger behaviour change. The application of a chosen BCT as part of a wider HH strategy is hypothesised to alter a specified behaviour determinant which in turn will change related behaviours. For example, watching videos promoting the importance of HH as part of a wider HH strategy is hypothesised to impact the nurse’s knowledge of the importance of practicing HH which in turn will lead to an increase in HHC.

2 We restricted our review to hospitals in high-income countries. The conditions of hospitals in low-income countries can be different than those to high-income countries, especially with respect to infection control measures and the infrastructure required for HH.

3 There were a total of 10 articles included; however, three of the articles described the same intervention (Huis et al.).

4 The OSCE is an exam where healthcare students examine and assess either real or simulated patients and are marked on their clinical skills performances.
