# Supplementary material for: The effect of behavioural interventions targeting hand hygiene practices among nurses in high-income hospital settings: a systematic review
Source: Public Health Rev. 2020 Dec 7;41:29. doi: 10.1186/s40985-020-00141-6 (PMC7720577; doi:10.1186/s40985-020-00141-6)
Supplement: Supplementary file 1 — Additional file 1. Search strings [file 40985_2020_141_MOESM1_ESM.docx]

## **APPENDIX 1-1: SEARCH STRINGS**

Search strings for the systematic literature review.

**SEARCH STRINGS**

1. handwashing

2. hand washing

3. hand wash

4. handwash

5. hand hygiene

6. 1 OR 2 OR 3 OR 4 OR 5

7. intervention*

8. program*

9. activit*

10. technique*

11. technolog*

12. protocol*

13. initiative*

14. campaign*

15. 7 OR 8 OR 9 OR 10 OR 11 OR 12 OR 13 OR 14

16. 6 AND 15

17. compliance*

18. observance

19. 17 OR 18

20. 16 AND 19

21. hospital*

22. healthcare

23. health care

24. healthcare environment*

25. health care environment*

26. healthcare setting*

27. health care setting*

28. 21 Or 22 OR 23 OR 24 OR 25 OR 26 OR 27

29. 19 AND 28

30. nurse*

31. nursing

32. 30 OR 31

33. 28 AND 32

34. Limit 33 to English and publications between 2002-2016
