# Supplementary material for: The effect of behavioural interventions targeting hand hygiene practices among nurses in high-income hospital settings: a systematic review
Source: Public Health Rev. 2020 Dec 7;41:29. doi: 10.1186/s40985-020-00141-6 (PMC7720577; doi:10.1186/s40985-020-00141-6)
Supplement: Supplementary file 2 — Additional file 2. Explanation of BCTS identified in studies [file 40985_2020_141_MOESM2_ESM.docx]

## **APPENDIX 1-2: EXPLANATION OF BCTs IDENTIFIED IN STUDIES**

Explanations of BCTS codes for *Fox et al.*

| ***Fox* et al. 2015** | | | | |
| --- | --- | --- | --- | --- |
| **Inputs and Implementation** | | | **Code and Reasoning** | |
| **Element** | **Activity** | **Components** |  |  |
| **Training** | Meeting | Members of nursing staff received verbal instructions from a study team member | **4.1 Instruction on how to perform a behaviour**  The intent of verbal instructions was to ensure that the nursing staff understood the correct hand hygiene technique. |  |
|  |  | Monitored for proper return demonstration of the patient hand hygiene protocol (PHHP) | **2.2 Feedback on behaviour**  Nurses demonstrated the PHHP and were monitored for proper return. ‘Monitor’ was interpreted as meaning that the nurses were watched closely for the purposes of ensuring performance aligned with the PHHP. It is assumed that feedback was provided.  **6.1 Demonstration of behaviour**  The team provided feedback to ensure performance aligned with the PHHP.  **8.1 Behavioural practice/rehearsal**  Nurses were prompted to practice the PHHP multiple times even though the actual intervention had yet to be implemented. The intention was to establish correct delivery of the PHHP. |  |
|  | EMR | Electronic medical record (EMR) triggers a timely reminder to perform the PHHP three times a day | **7.1 Prompts/cues**  As nurses are focused on other duties (such as charting), they are reminded to perform the PHHP through prompts on the EMR.  **8.3 Habit formation**  The repeated prompting to perform the PHHP leads to the continuous repetition of performing the PHHP. |  |
|  |  | Prompt in the EMR also requires nurses to document HH protocol adherence | **2.3 Self-monitoring of behaviour**  Nurses are required to report whether or not they performed the PHHP.  **15.3 Focus on past success**  By reporting one’s compliance with the PHHP, nurses are required to reflect and focus on the action—the performance of the PHHP—that has just been completed. |  |
|  | Room visits | PHHP  cleaning patients hands | **8.6 Generalization of target behaviour**  In healthcare settings, attention regarding the promotion of HH is focused mainly on HCWs. In this intervention, the promotion of HH is focused on the patients.  **13.2 Framing/reframing**  The act of performing the PHHP is framed in terms of patient care. The patient is at the centre of this intervention—nurses’ own HH behaviour is not emphasized, only adherence to the PHHP.  **13.5 Identity associated w/changed behaviour**  **Reasoning:** By performing the PHHP, nurses create bonds with their patients. The nurses adopt the identity of nurturer. Therefore, the role of the nurse is expanded, and as such identity is impacted. |  |

| ***Erasmus* et al. 2010** | | | |
| --- | --- | --- | --- |
| **Inputs and Implementation** | | | **Code and Reasoning** |
| **Element** | **Activity** | **Components** |  |
| **Structured Interview** | Education and reflection | Importance of hand hygiene | **5.1 Information about health consequences**  It is assumed that in discussing the importance of HH, the consequences of not performing HH would also be included. |
|  |  | Rated self-compliance | **1.6 Discrepancy between current behaviour and goal**  By asking the participants to reflect on their own HH behaviour through rated self-compliance, participants must consider their own behaviour and compare it to the desired behaviour learned about in the previous component. |
|  |  | Preferred methods of hand hygiene | **Unable to code**  **Reasoning:** The description of this component is vague and unable to be coded. |
|  |  | Possible barriers encountered | **1.2 Problem solving**  An analysis of barriers to performing HH were identified. |
|  | Implementation intention | Action plans | **1.4 Action planning**  plans for performing HH in specific contexts were created by the participants. |
|  | Coping Plan | Coping planning | **1.2 Problem solving**  Participants anticipated and planted alternatives for moments when they were unable to execute their own action plan. This is coping planning. |

| ***Stock* et al. 2015** | | | |
| --- | --- | --- | --- |
| **Inputs and Implementation** | | | **Code and Reasoning** |
| **Element** | **Activity** | **Components** |  |
| **12- 45 minute sessions of ‘training’ over 1.5 days** | First part: Initial objective structured clinical examination (OSCE) via stations | Evaluate one of the key hygiene skills defined previously | **Unable to code**  The description of this component is vague and unable to be coded. |
|  |  | Give participants the chance to reflect on their hygiene and communication skills^[[1]](#footnote-1)^ | **1.6 Discrepancy between current behaviour and goal**  By asking the participants to reflect on their own HH behaviour and communication skills, participants must consider their own behaviour and compare it to the desired behaviour.  **15.2 Mental rehearsal of successful performance**  Participants were presented new information and asked to reflect on their own behaviour as well as on the new material presented. It is assumed that in doing so, the participants imagined performing the behaviour successfully. |
|  | Second part: a combined theoretical and practical learning session | Lectures and role-play to train communication and feedback methods | **8.1 Behavioural practice/rehearsal**  The lectures and role-play allow for participants to repeatedly practice the behaviour in a simulated context. |
|  |  | Reflection and evaluation of communication abilities | **2.3 Self-monitoring of behaviour**  The participants monitor their behaviour through constant reflection and evaluation. What sets this component apart from the reflection component in the initial OSCE phase is the word ‘evaluate’. Participants evaluate their own abilities. |
|  |  | Quality management in hospital hygiene | **Unable to code**  **Reasoning:** The description of this component is vague and unable to be coded. |
|  |  | Methods to address barriers to hygiene when communicating with peers and superiors | **1.2 Problem solving**  Identifying and then creating strategies to address barriers is problem-solving. |
|  | Third part: a combined theoretical and practical simulation training | Theoretical information | **Unable to code**  The description of this component is vague and unable to be coded. |
|  |  | Simulation training in hygiene skills in different situations. Participants were encouraged to ask questions and practiced hygiene skills under the supervision of the infection control nurse | **2.2 Feedback on behaviour**  Participants demonstrated HH skills under the supervision of the infection control nurse. Participants were encouraged to ask questions. As such, it is assumed that the nurse provided feedback.  **4.1 Instruction on how to perform the behaviour**  The simulation training is a form of skills training.  **6.1 Demonstration of the behaviour**  The simulation requires participants to learn how to act in certain situations. It is assumed that such behaviour has been demonstrated by others, such as the infection control nurse. Also, participants could regard the infection control nurse as a role model, and as such, model their own behaviour accordingly.  **8.1 Behavioural practice/rehearsal**  Participants demonstrated HH skills in different situations under the supervision of the infection control nurse through the simulation training. It is assumed that the simulations were repeatedly run with participants practicing multiple times.  **8.3 Habit formation**  Participants demonstrated HH skills in different situations under the supervision of the infection control nurse through the simulation training. It is assumed that the simulations were repeatedly run with participants practicing multiple times. |
|  | Fourth part: final OSCE evaluation | OSCE assessment was repeated to evaluate improvements in  hygiene skills | **2.7 Feedback on outcome(s) of behaviour**  The feedback provided is not specifically about performing the act of HH, but rather on the outcome of increased HH practice. |

| ***Harne-Britner* et al. 2011** | | | |
| --- | --- | --- | --- |
| **Inputs and Implementation** | | | **Code and Reasoning** |
| **Element** | **Activity** | **Components** |  |
| **Control** | Self-study module | Hand washing educational self-module with additional education about microorganisms | **4.1 Instruction on how to perform the behaviour**  The educational module covers various aspects of handwashing. It is assumed that the module covers how to perform HH behaviour.  **5.1 Information about health consequences**  The educational module covers various aspects of handwashing. It is assumed that the module covers information about health consequences.  **6.1 Demonstration of the behaviour**  The educational module covers various aspects of handwashing. It is assumed that the module demonstrates how to perform HH behaviour. |
| **Positive reinforcement**^[[2]](#footnote-2)^ | Individual sticker chart | Staff members placed a sticker beside peer’s name as they witnessed HH being performed | **6.2 Social comparison**  Stickers are awarded by peers. As a result, the staff members pays more attention to others’ HH performance. This in turn allows for individuals to compare their own HH performance to that of the larger group.  **6.3 Information about others’ approval**  Stickers are awarded by peers. As a result, the staff members pays more attention to others’ HH performance. |
|  |  | Staff member with most stickers received a reward | **10.8 Incentive (outcome)**  Staff members were informed that the nurse with the most stickers would receive a reward.  **10.10 Reward (outcome)**  The staff member with the most stickers received a reward such as movie tickets and gift cards from local grocery stores, gas stations, and restaurants. |
|  |  | Staff member with most stickers also received public recognition | **10.4 Social reward**  The staff member with the most stickers received public recognition, which is a social reward.  **10.5 Social incentive**  Staff members were informed that the nurse with the most stickers would be publicly recognized. |
|  | Unit reward | Unit rewarded a pizza party if HHC goal reached | **1.3 Goal setting (outcome)**  The unit set a HHC goal to which it strived to work towards accomplishing.  **10.8 Incentive (outcome)**  The unit was informed that there would be a reward if the goal had been reached.  **10.10 Reward (outcome)**  If the unit reached its goal, it would receive a pizza party. |
|  | Monthly quality meetings | Feedback on infection and HHC rates | **2.7 Feedback on outcome(s) of behaviour**  The infection and HHC rates were shared during the monthly quality meetings. This provided feedback on how the unit was performing. |
|  |  | Adherence goal-setting | **1.3 Goal setting (outcome)**  A HHC goal was set and agreed upon. |
| **Risk of Nonadherence**^[[3]](#footnote-3)^ | Posters | Cartoon bug posters stating “I am on your hands heading to your patients!” were placed around the unit | **5.1 Information about health consequences**  This poster provided information visually about the consequences of not performing HH.  **7.1 Prompts/cues**  This poster was placed around the unit in order to prompt the staff members to practice HH. |
|  |  | An additional poster of the agar plates about organisms found on the hands was also placed on walls. | **5.1 Information about health consequences**  This poster provided information visually about the consequences of not performing HH.  **7.1 Prompts/cues**  This poster was placed around the unit in order to prompt the staff members to practice HH. |
|  | Agar plate culture of hands | The results of hand cultures done on the unit were shared with the unit | **5.2 Salience of consequences**  The agar plate culture component was specifically designed to emphasize the consequences of not practicing HH. This component of the intervention is memorable because it is different from the rest (posters, self-study module, etc.). |

| ***Huis* et al. 2012** | | | |
| --- | --- | --- | --- |
| **Inputs and Implementation** | | | **Code and Reasoning** |
| **Element** | **Activity** | **Components** |  |
| **State-of-the-art strategy** | Meetings | Distribution of educational material/written information (leaflet) about HH that contained: the importance of HH; misconceptions about ABHR; theory and practical indications for the use of HH | **4.1 Instruction on how to perform the behaviour**  The material included the indications for the use of HH in addition to general information about HH. It is assumed that the material advised nurses on how to perform the behaviour.  **5.1 Information about health consequences**  It is assumed that in discussing the importance of HH, the consequences of not performing HH would also be included |
|  |  | Notification of website: Educational material/written information about HH; knowledge quiz with feedback; nursing ward with highest number of visitors to the website was rewarded | **4.1 Instruction on how to perform the behaviour**  The website contained educational material on HH. It is assumed that this advises nurses on how to perform the behaviour.  **5.1 Information about health consequences**  It is assumed that in discussing the importance of HH, the consequences of not performing HH would also be included.  **10.6 Non-specific incentive**  Nurses were informed that a reward would be given to the unit with the highest number of visits to the website. It is assumed from the study that the nurses did not know what the award was.  **14.6 Situation-specific reward**  The reward was specifically given to the unit with the highest number of visits to the website. This was a single event as compared to the continuous monitoring of HHC which is comprised of many smaller HH events. |
|  |  | Bar charts of HH rates of every nursing ward were sent to the ward manager twice. It also included a comparison of ward performance to hospital performance | **2.7 Feedback on outcome(s) of behaviour**  The bar charts provide feedback on the HHC rates of the unit.  **6.2 Social comparison**  The bar charts also drew attention to other units’ performances which allowed for each unit to compare its own performance to other units in the hospital. |
|  | Hospital-wide campaign launch | Practical demonstrations | **4.1 Instruction on how to perform the behaviour**  It is assumed that the practical demonstrations provided instruction on HH performance.  **6.1 Demonstration of the behaviour**  The practical demonstrations provided an observable sample of HH performance. |
|  | Newsletter | Interviews and messages | **Unable to code**  **Reasoning:** The description of this component is vague and unable to be coded. |
|  | Ad hoc | General reminders by opinion leaders/ ward management | **9.1 Credible source**  Opinion leaders and ward management are considered to be credible sources as “they pull weight” and are influential. |
|  | Environment modification | Screening and if necessary adapting products and appropriate facilities | **12.1 Restructuring the physical environment**  Although vague, it is assumed from the description that when necessary there was a change to the physical environment in order to facilitate performance of HH.  **12.5 Adding objects to the environment**  Although vague, it is assumed from the description that when necessary there was a change to the physical environment in order to facilitate performance of HH. |
|  |  | Posters that emphasized the importance of HH,  particularly ABHR | **7.1 Prompts/cues**  These posters were places around the unit in order to prompt the staff members to practice HH. |
| **Team and leaders-directed strategy**^[[4]](#footnote-4)^ | Meetings with ward management | Ward managers share experiences and discuss difficulties with one another | **1.2 Problem solving**  Ward managers discussed difficulties with one another. It is assumed that the ward managers helped one another develop strategies to address the difficulties.  **3.2 Social support (practical)**  Ward managers discussed experiences and difficulties with one another. It is assumed that the ward managers helped one another develop strategies to address the difficulties. |
|  | Three interactive team sessions (1-1.5 hour) | Team members explore their own HH behaviour | **1.6 Discrepancy between current behaviour and goal**  By asking the participants to reflect on their own HH behaviour, participants must consider their own behaviour and compare it to the desired behaviour. |
|  |  | Team members analyse barriers and facilitators | **1.2 Problem solving**  Team members identified barriers and facilitators to the performance of HH. |
|  |  | Team members formulate improvement activities | **1.4 Action planning**  Team members create improvement activities to aid in the performance of HH. |
|  |  | Team members make commitment to achieve a substantial increase in HHC | **1.9 Commitment**  Team members indicated a commitment to increasing HHC rates. |
|  |  | Ward manager presents the HHC rates of the previous period | **2.7 Feedback on outcome(s) of behaviour**  The ward manager provided feedback on the previous period’s HHC performance. |
|  |  | Team members discuss the rates by asking a series of questions reflecting on their behaviour (and how it could have affected the outcome). | **1.2 Problem solving**  Team members identified barriers and facilitators to the performance of HH.  **1.6 Discrepancy between current behaviour and goal**  The attention of the team members was drawn to the discrepancies between their current HH behaviour and the set outcome goal (HHC rate).  **15.3 Focus on past success**  Team members had to think about their own past performance, which did include past successes. |
|  | Ad hoc | Nurses address each other in case of undesirable HH behaviour | **3.2 Social support (practical)**  Nurses advise one another on their HH performance.  **6.3 Information about others’ approval**  The act of addressing another in terms of undesirable HH behaviour showcases what other people think. |
|  |  | Modelling by informal leaders at the ward: informal leaders demonstrated good HH behaviour; informal leaders modelled social skills of team members in addressing HH behaviour of colleagues; informal leaders instructed and stimulated their colleagues in providing good HH behaviour | **3.2 Social support (practical)**  Informal leaders advised and provided help in regard to the performance of HH.  **4.1 Instruction on how to perform the behaviour**  Informal leaders advised on how to perform good HH.  **6.1 Demonstration of the behaviour**  Informal leaders provided an observable sample of performing HH behaviour in addition to how to address HH behaviour of colleagues. |

| ***Boyce* et al. 2019** | | | |
| --- | --- | --- | --- |
| **Inputs and Implementation** | | | **Code and Reasoning** |
| **Element** | **Activity** | **Components** |  |
| **Automated HH Monitoring System** | The AHHM system was installed in the hospital | Sensors were placed in dispensers and in the entry ways of the patient rooms | **2.7 Feedback on outcome(s) of behaviour**  The monitoring system provided HH rates.  **12.5 Adding objects to the environment**  The AAHHM system was added to the environment in order to help facilitate practicing HH. |
| **Goal Setting** | Nursing units team members set goals | Goals were set for improved HH performance rates. As goals were met, units celebrated their achievements and set new goals | **1.3 Goal setting and outcome**  The team members agreed on a goal.  **1.7 Review outcome goals**  The team members reviewed outcome goals and modified goals in light of achievement.  **2.7 Feedback on outcome(s) of behaviour**  The team members monitored and provided informative feedback on outcome.  **10.4 Social reward**  When goals were met, there was a verbal reward in the form of positive reinforcement. |
| **Frontliine Ownership Initiative (Intervention 1)** | An expert visited the hospital three times to assist in implementing FLO | FLO is aimed at owning the problem and to deepen awareness thereby prompting a solution | **1.2 Problem solving**  By turning to an expert and trying to implement FLO, the hospital is trying to address the low HHC rates by owning the HH problem and analysing ways to move forward.  **1.4 Action Planning**  There is detailed planning of performance of the behaviour. |
| **Support by Hospital Leadership**  **(Intervention 2)** | Hospital leadership sent a delegate to another hospital who had success in sustaining increased HHC. | Nurses, infection preventionist, and the vice president of medical affairs travelled to another hospital to learn about their successful multimodal HH campaign; discussed methods for analysing AHHMS data and additional promotional activities | **6.2 Social comparison**  By drawing comparison to the other hospital’s performance, the study hospital is able to compare their own performance.  **9.3 Comparative imagining of future outcomes**  Prompt or advise the imagining and comparing of future outcomes of changed versus unchanged |
| **DO NO HARM team HH audit** | Covert direct observations of HHC. | Members of the HH audit team covertly conducted direct observations of HHC upon entry and exit of patient rooms on all nursing units | **2.5 Monitoring of behaviour by others without feedback**  The Do No Harm team conducted covert HH audits. |
| **Toyota Kata (intervention 3)** | Trainings for hospital leadership. | Mandatory training for management and leadership staff | **Unable to code**  **Reasoning: T**he description of the training is vague and is unable to be coded. |
|  | Develop institutional commitment | Through meetings and trainings, the hospital was able to create awareness, which allowed for ownership of the HH problem and further displayed the hospital’s commitment to tackling this problem | **1.9 Commitment**  The hospital is indicating a commitment to changing HH behaviour. |
|  | HH sheriff | On each unit, one person every day was assigned to wear a “HH Sheriff” badge and reminded personnel to perform HH | **2.2 Feedback on behaviour**  The sheriff provides real-time feedback when encouraging personnel to practice HH.  **7.1 Prompts/cues**  Introduce or define environmental or social stimulus with the purpose of prompting or cueing the behaviour  **13.1 Identification of self as role model**  HH sheriff comments on others’ behaviours; their behaviour is an example to others. |
|  | Sharing HH rates | HH rates were reported at shift huddles and safety huddles; the rates were posted in the staff lounges and shared with hospital leadership | **1.6 Discrepancy between current behaviour and goal**  By sharing HHC rates, the current HH behaviour can be compared the hospitals’ set outcome goals.  **2.7 Feedback on outcome(s) of behaviour**  Sharing the HHC rates allows nurses to see where the unit’s rates are that day/week. |
|  | Coaching nurses | Healthcare personnel were coached when HHC decreased | **Unable to code**  **Reasoning:** The description of how or what personnel were coached on is vague and is unable to be coded. |

| ***Stella et al*. 2019** | | | |
| --- | --- | --- | --- |
| **Inputs and Implementation** | | | **Code and Reasoning** |
| **Element** | **Activity** | **Components** |  |
| **Eye image intervention** | Posters | Placards depicting the human eyes with reminder to “clean hands on entry and exit” were displayed above soap and ABHR dispensers on exterior of patient rooms; they were rotated with the control image of mountains | **4.1 Instruction on how to perform behaviour**  The posters specifically mention performing HH upon entry and exit.  **7.1 Prompts/ cues**  The posters were placed around the unit in order to prompt the staff members to practice HH.  **16.1 Imaginary punishment**  Advise to imagine performing the **unwanted** behaviour in a real-life situation followed by imagining an unpleasant consequence |
| **Social norm message intervention** | Posters | Placards depicting images of several healthcare professionals with a message encouraging compliance with social norms. A slogan included reference to being the ‘dirty one’. An authoritative agent was also referenced as sponsoring the poster | **6.2 Social comparison**  On the poster are other HCWs. This will draw the attention of the staff members to others’ HH performance and allow comparison with the person’s own performance.  **7.1 Prompts/ cues**  The posters were placed around the unit in order to prompt the staff members to practice HH.  **9.1 Credible source**  Having the hospital as the sponsor and including HCWs who appear knowledgeable and assured lead to the message seeming like it comes from a credible source.  **11.2 Reduce negative emotions**  Advise on ways of reducing negative emotions to facilitate performance of the behavior [don’t be dirty one]. |

1. It is difficult to code ‘reflection.’ [↑](#footnote-ref-1)
2. The self-study module is also included in the positive reinforcement intervention. [↑](#footnote-ref-2)
3. The self-study module is also included in the risk of nonadherence intervention. [↑](#footnote-ref-3)
4. The team and leaders-directed strategy includes all the activities and components of the state-of-the-art strategy. [↑](#footnote-ref-4)
