# Supplementary material for: The effect of behavioural interventions targeting hand hygiene practices among nurses in high-income hospital settings: a systematic review
Source: Public Health Rev. 2020 Dec 7;41:29. doi: 10.1186/s40985-020-00141-6 (PMC7720577; doi:10.1186/s40985-020-00141-6)

APPENDIX 1-3: LOGIC MODELS DEVELOPED FOR STUDIES

Logic models inferred for each study with the nominated BCTS and the theory of change behind each intervention.

Figure A2- 1: Logic model for *Stock et al.*

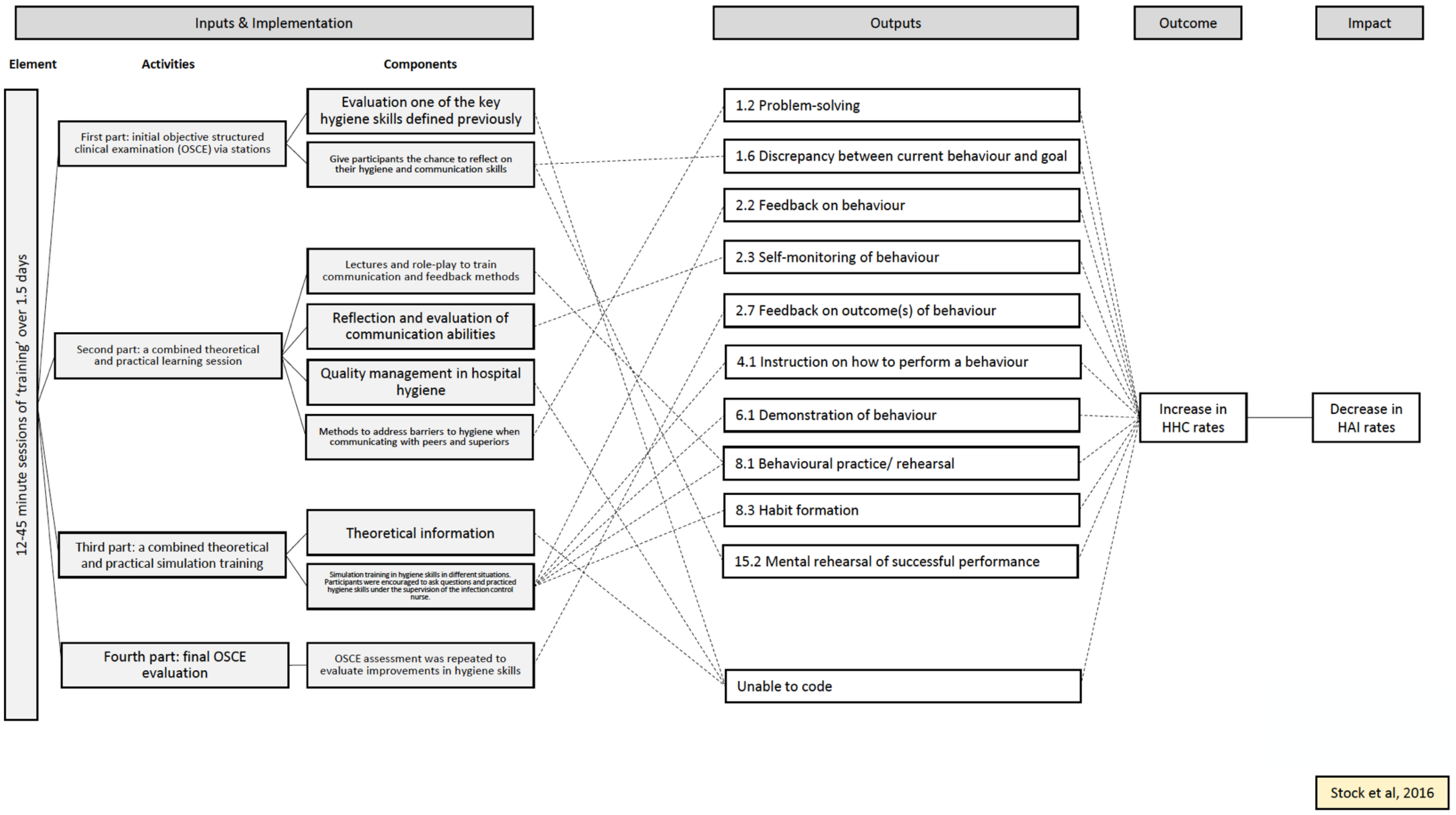

Figure A2- 2: Logic model for *Erasmus et al.*

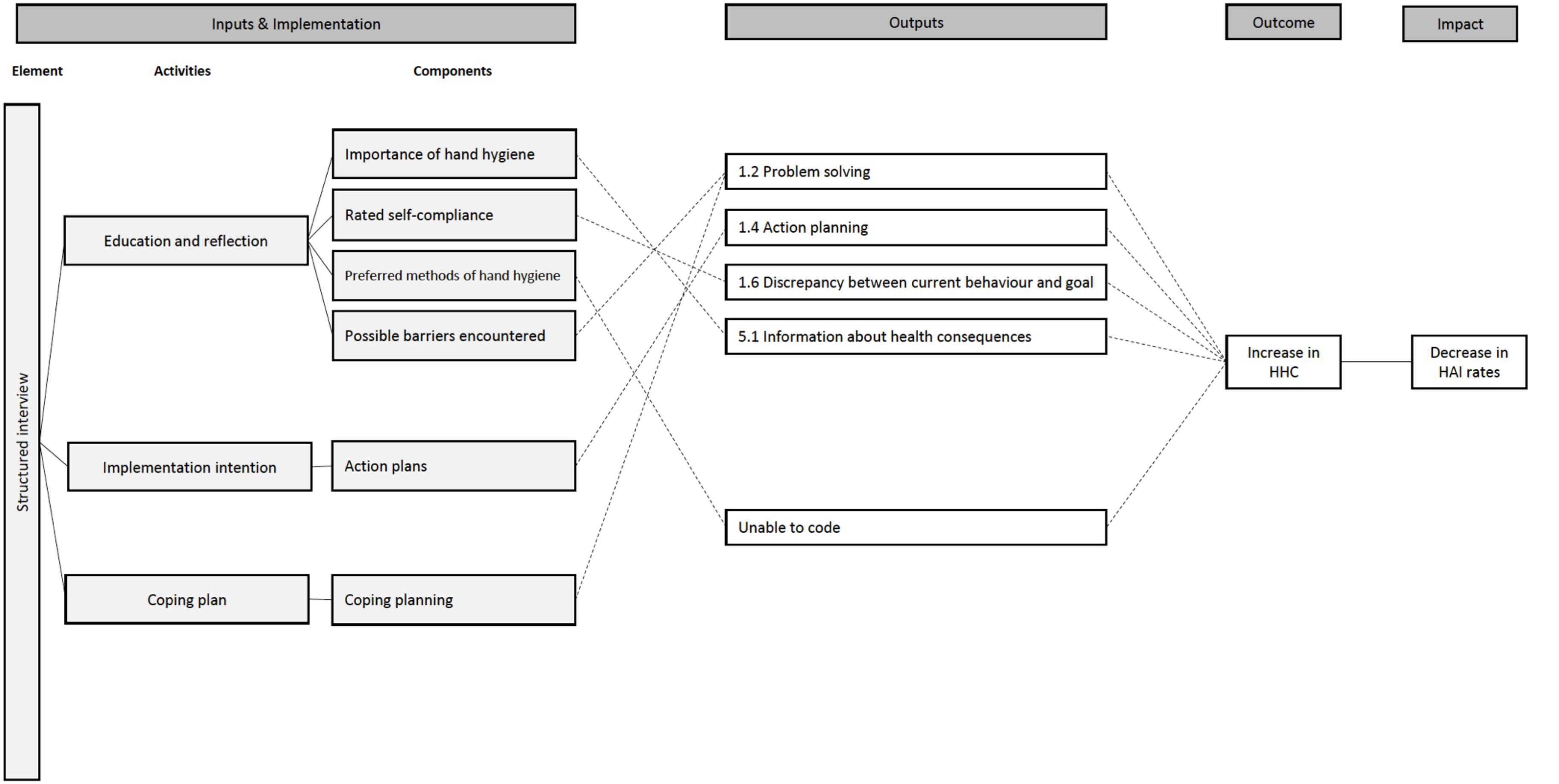

Figure A2- 3: Logic model for *Fox et al.*

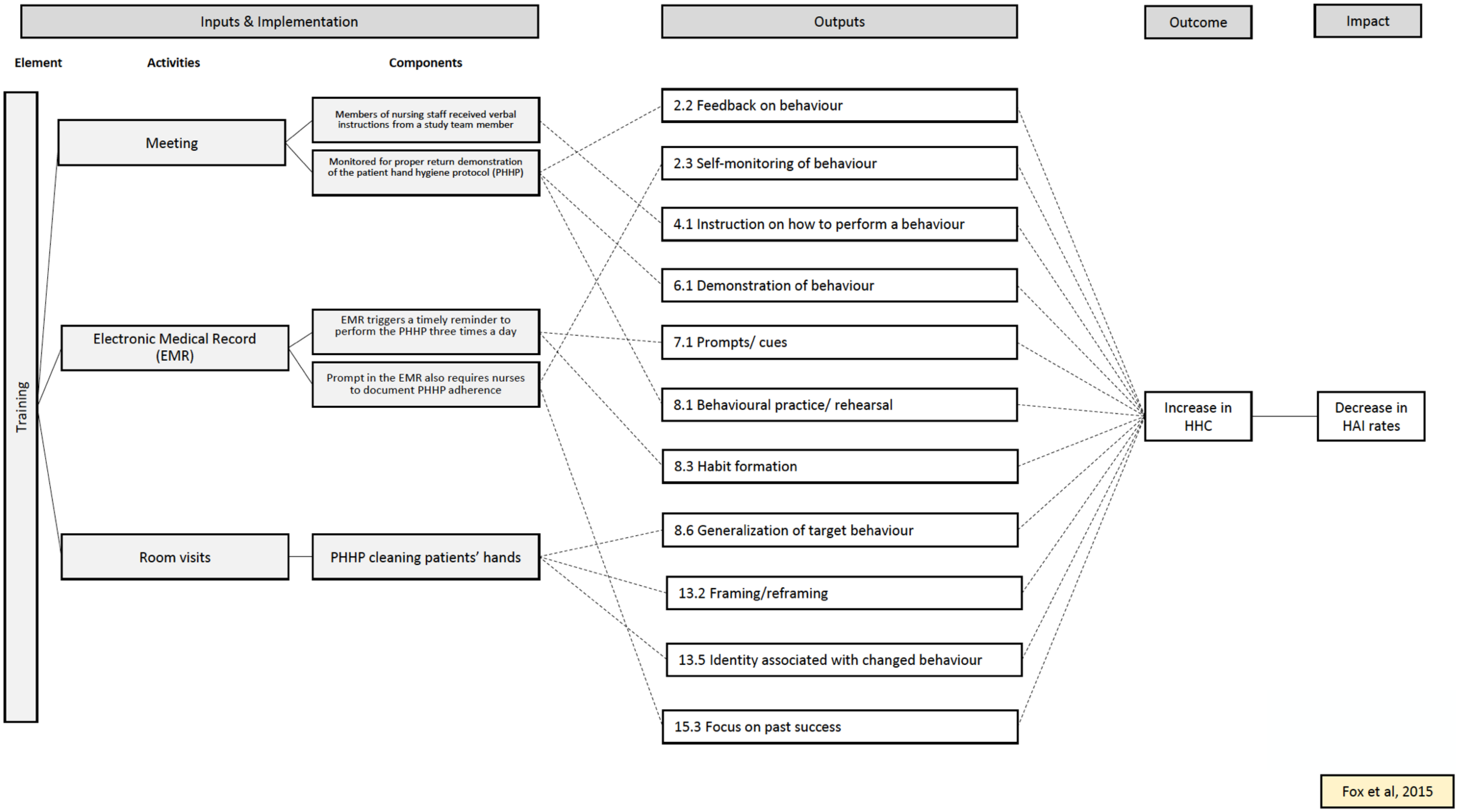

Figure A2- 4: Logic model for *Harne-Britner et al* control.

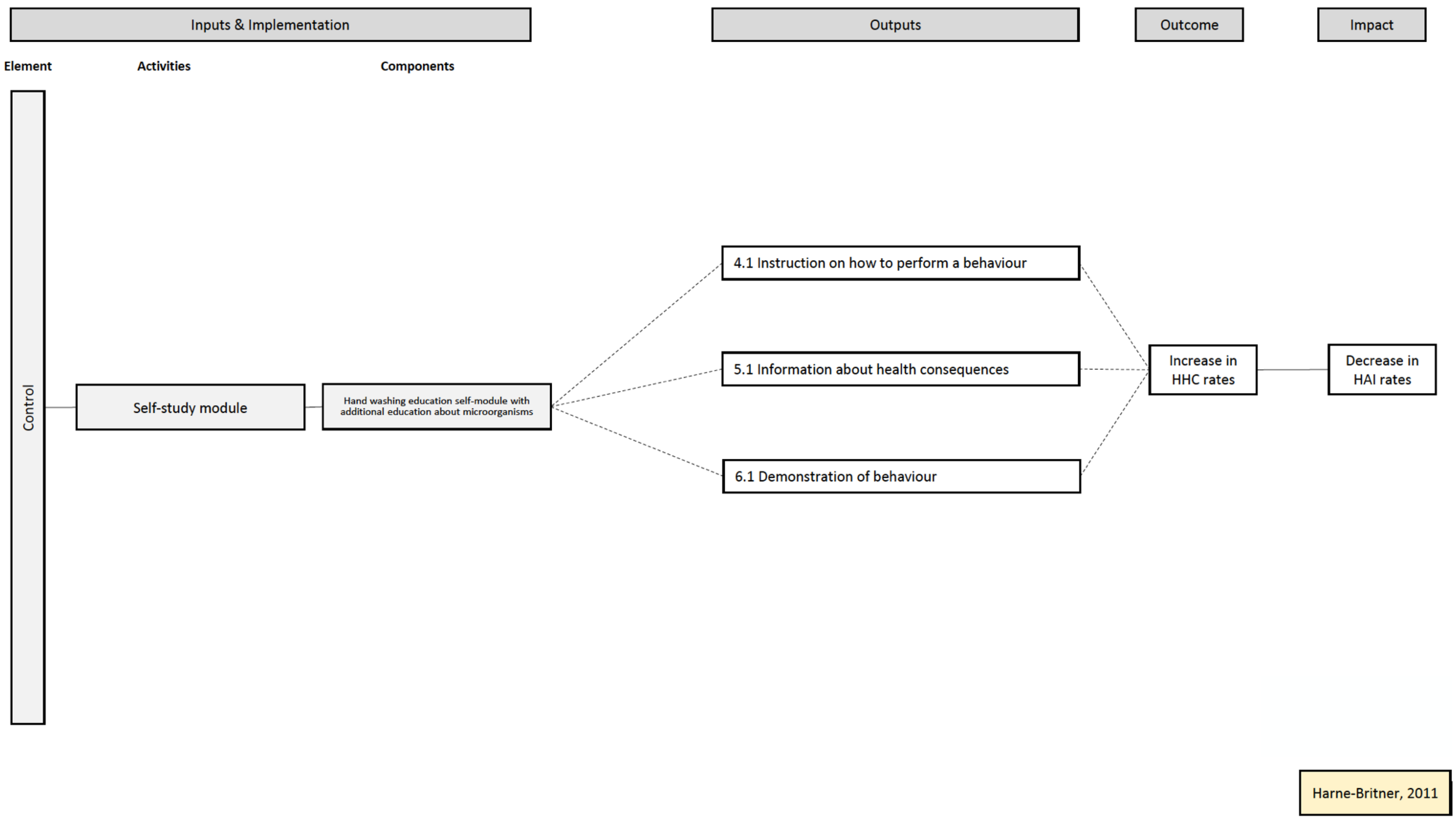

Figure A2- 5: Logic model for *Harne-Britner et al.* risk of nonadherence

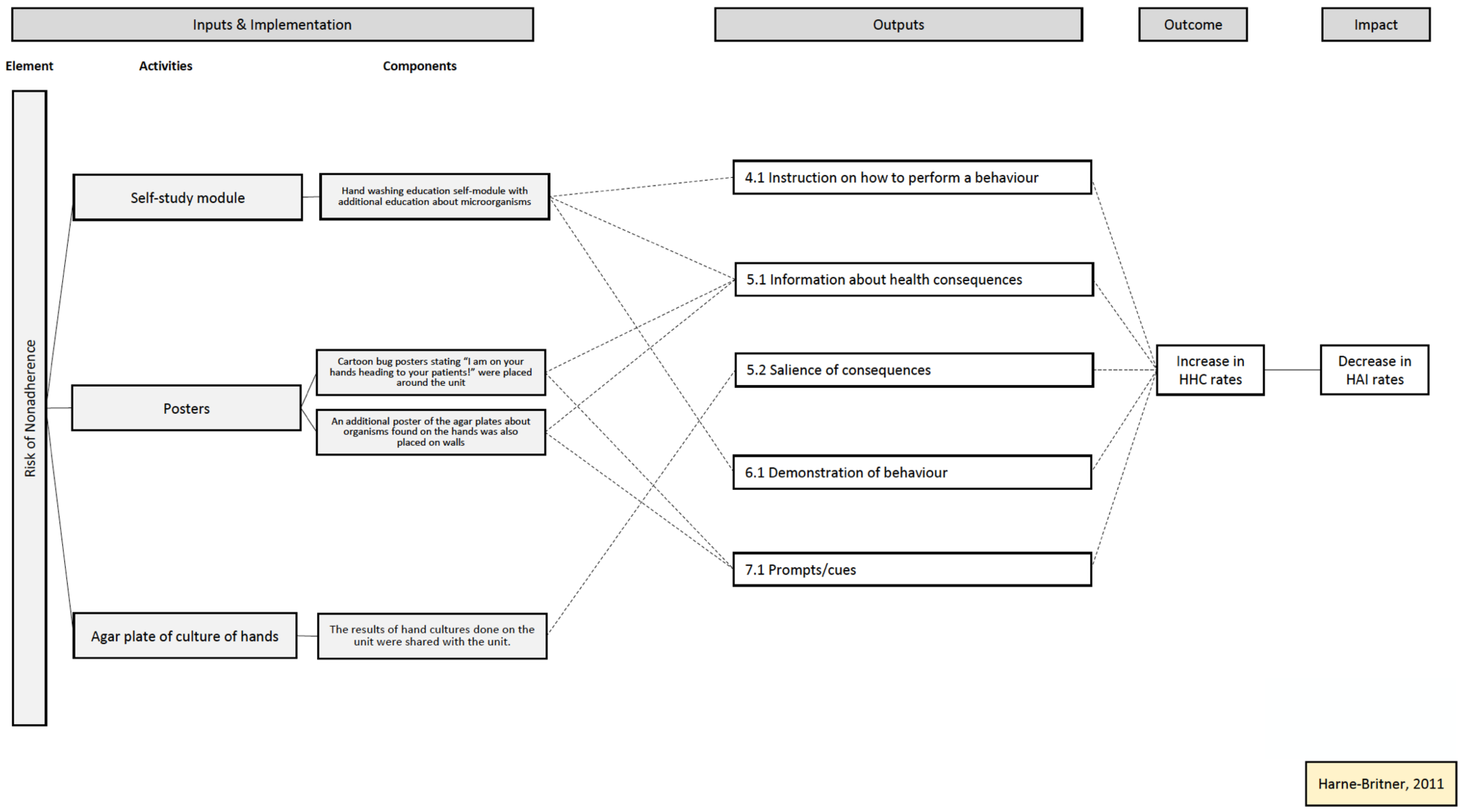

Figure A2- 6: Logic model for *Harne-Britner et al.* positive reinforcement

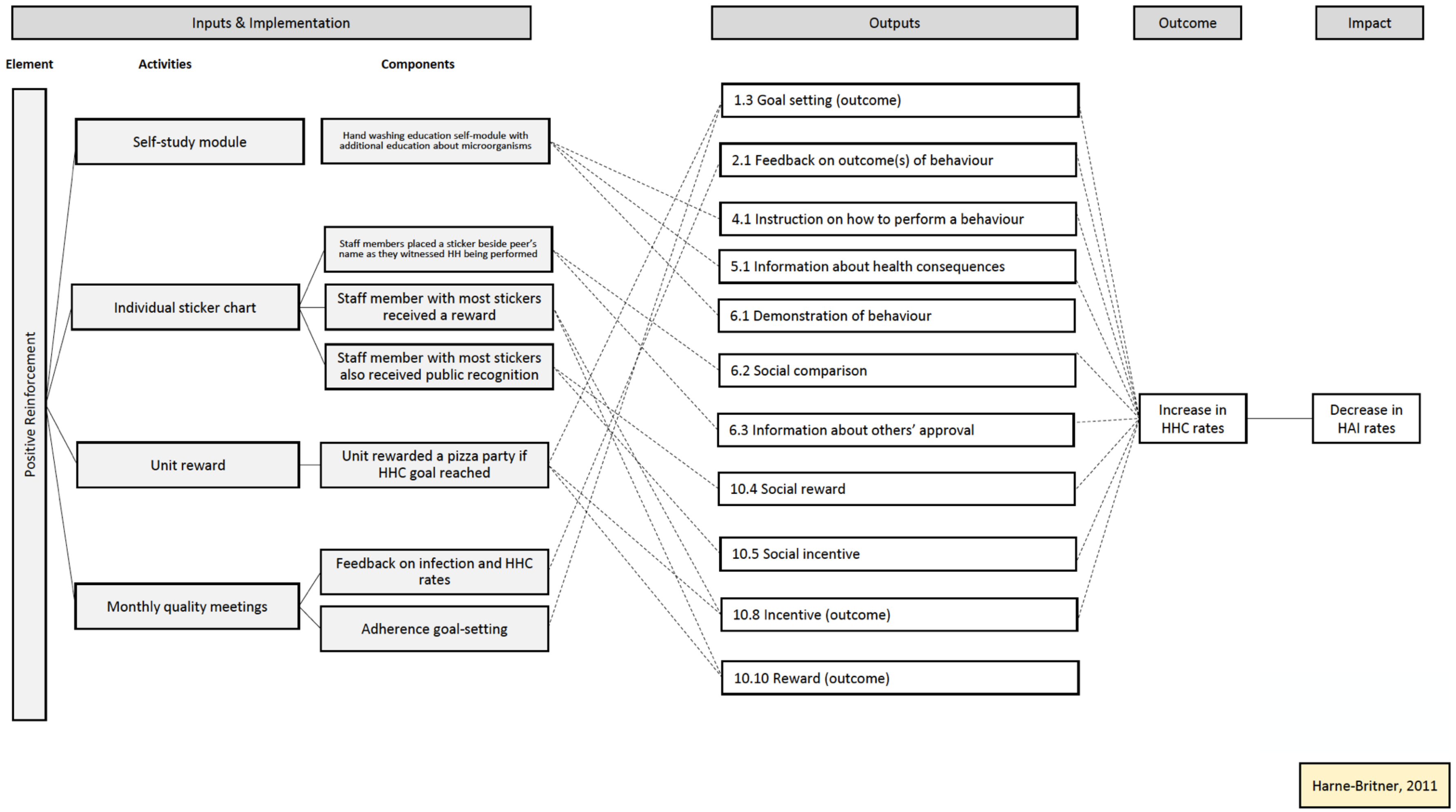

Figure A2- 7: Logic model for *Huis et al.* state-of-art-strategy

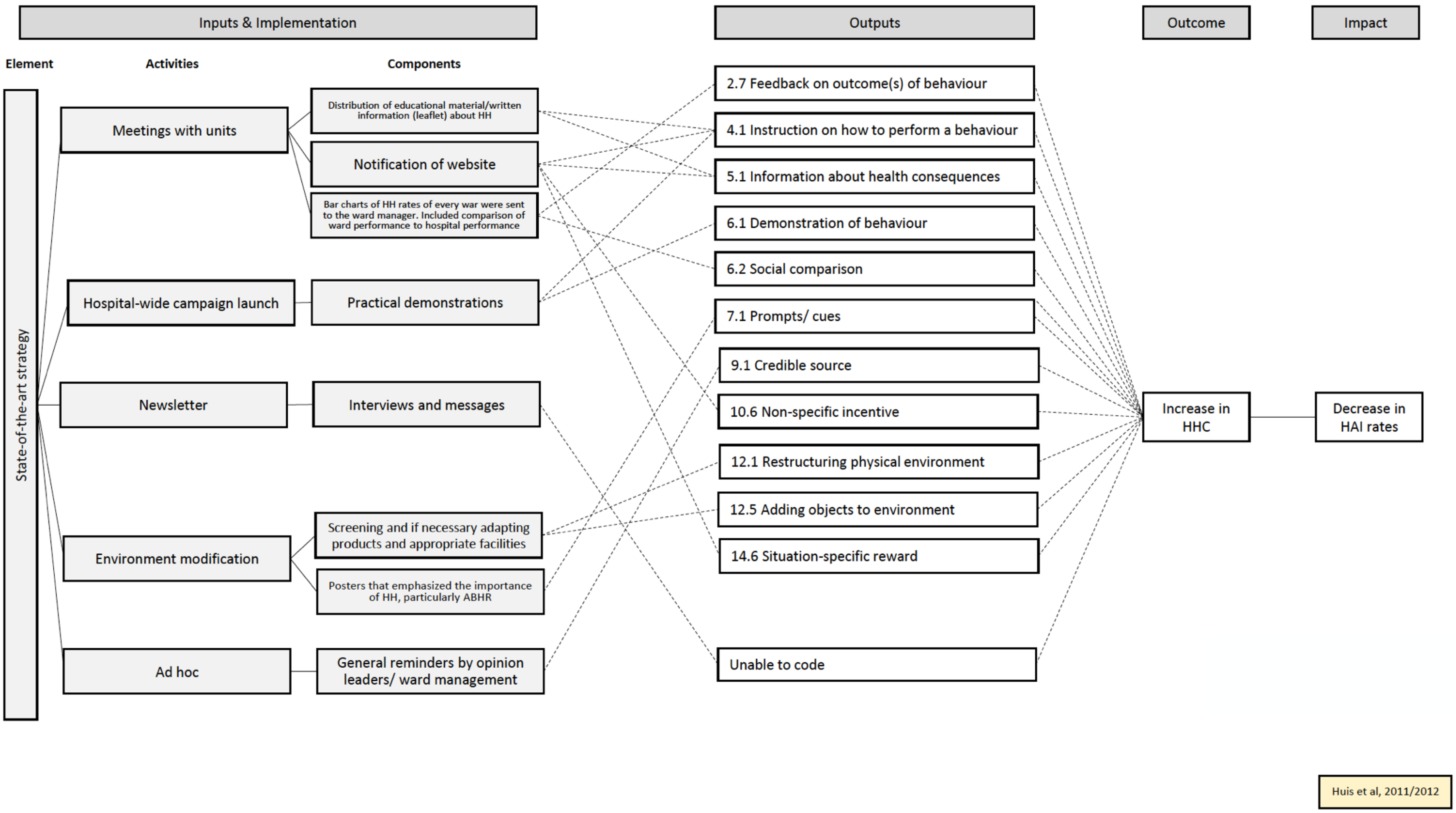

Figure A2- 8: Logic model for *Huis et al.* team strategy

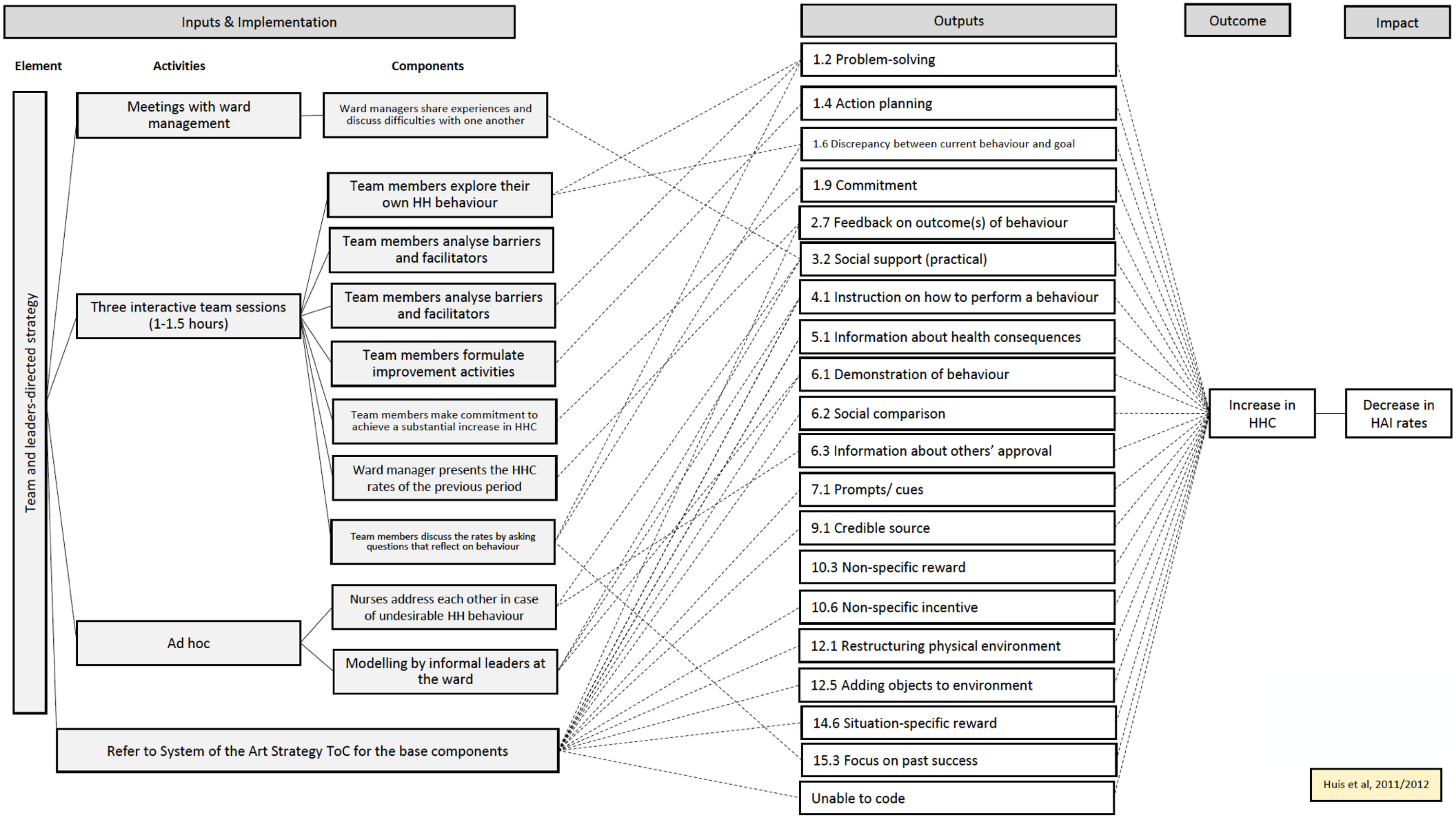

Figure A2- 9: Logic model for *Stella et al.* intervention

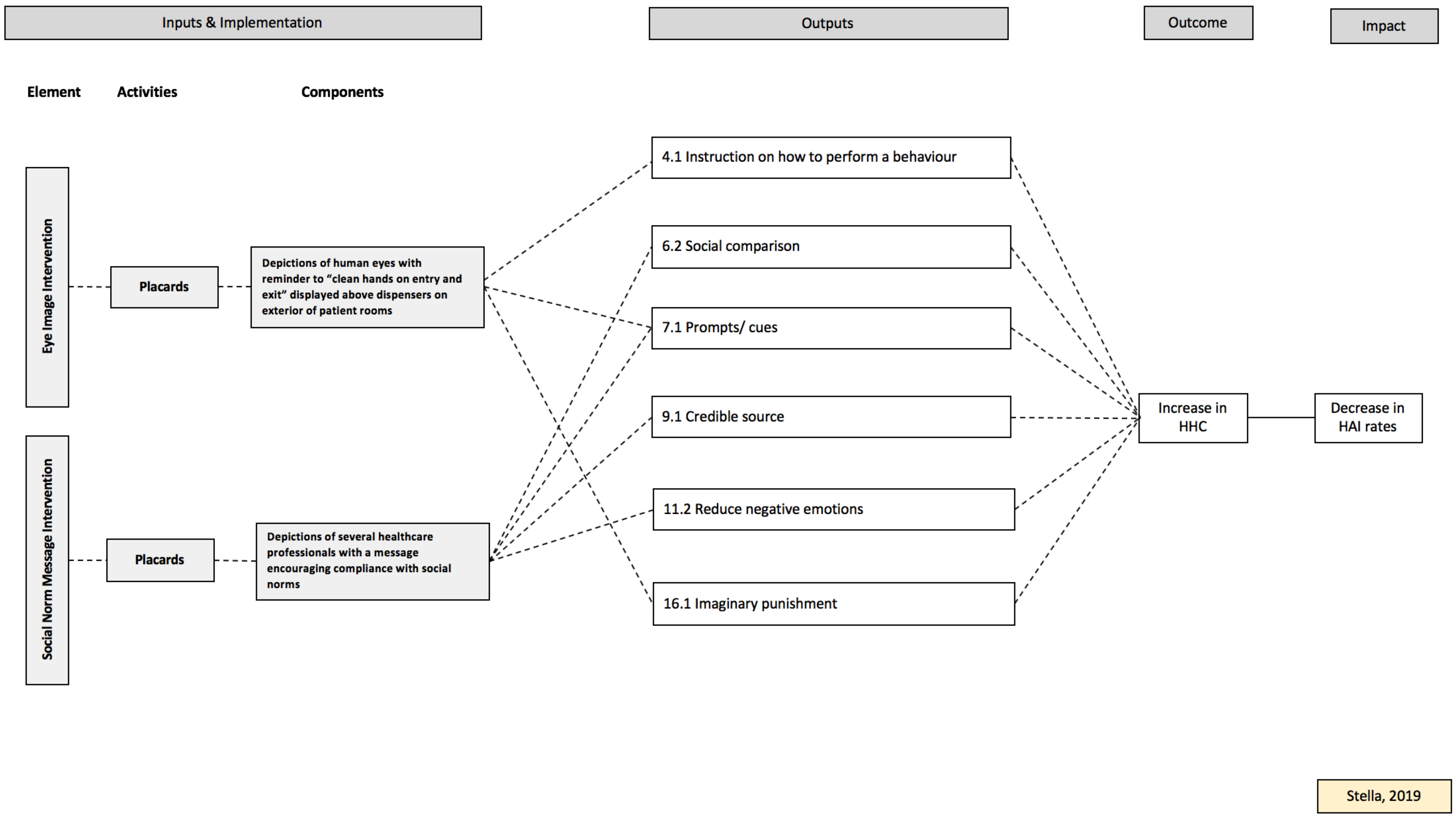

Figure A2- 10: Logic model for *Boyce et al.* interventions 1 and 2

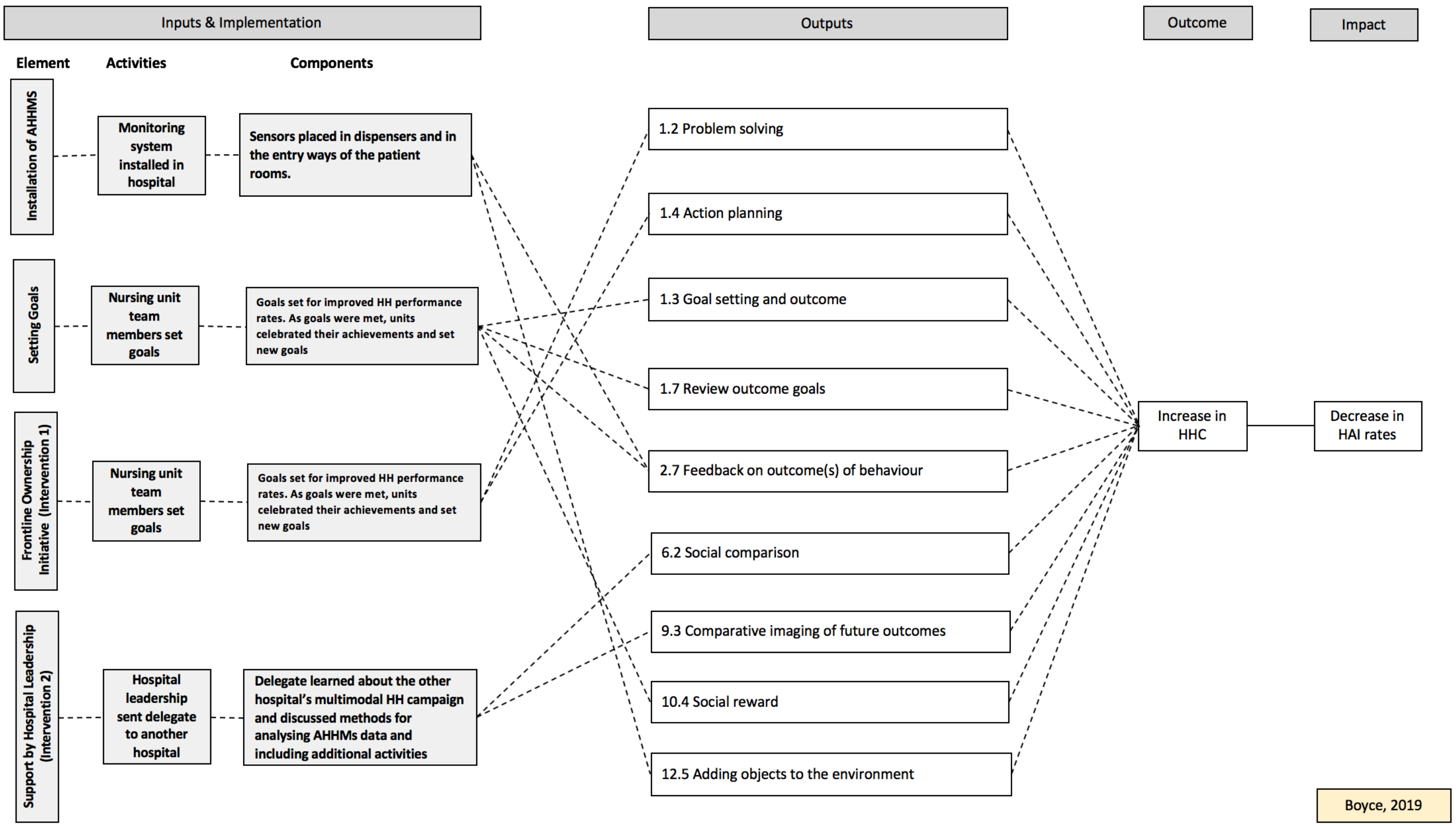

Figure A2- 11: Logic model *Boyce et al.* intervention 3

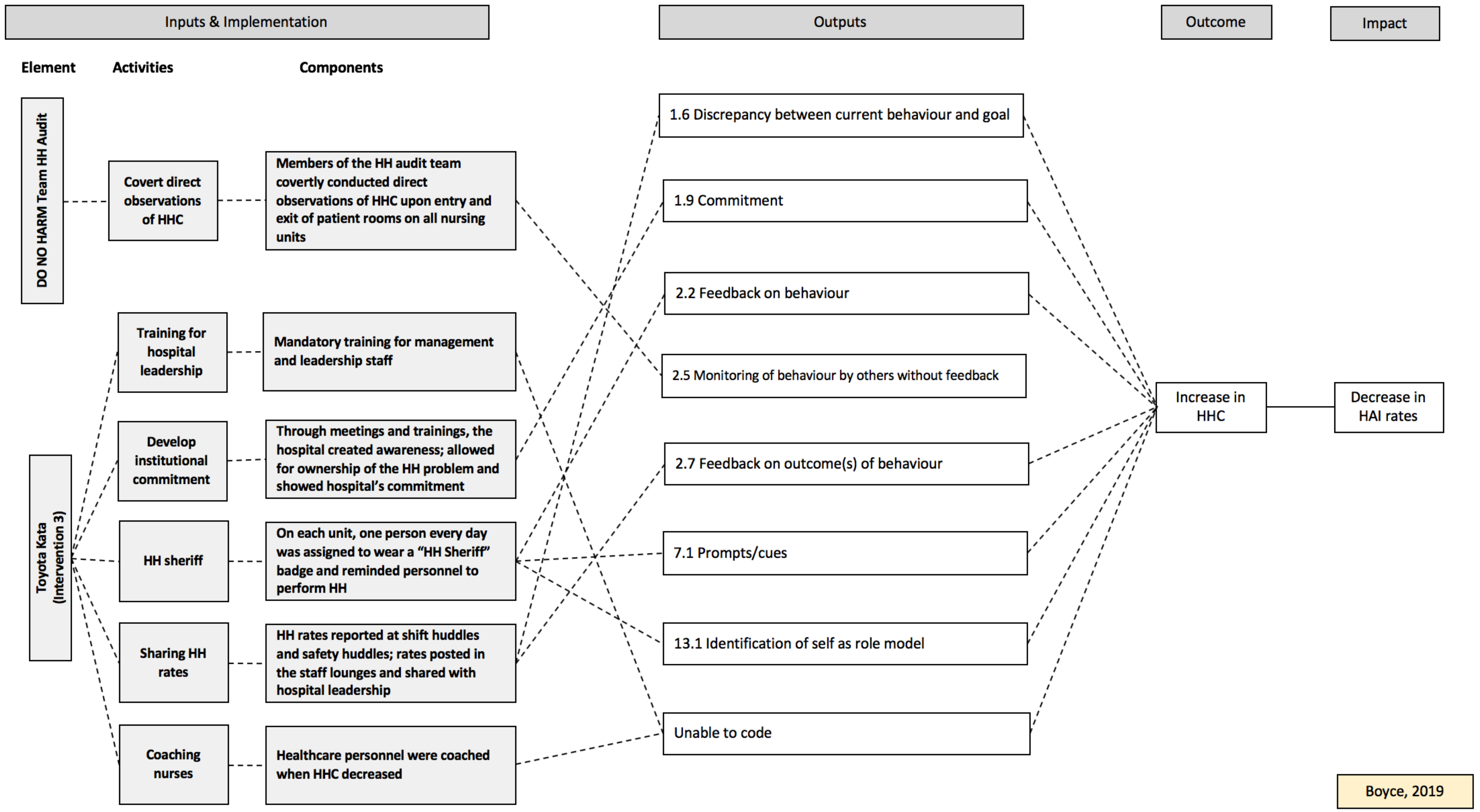

Supplement: Supplementary file 3 — Additional file 3. Logic models developed for studies [file 40985_2020_141_MOESM3_ESM.pdf]
